# Impact of HIV-ART on the restoration of Th17 and Treg cells in blood and female genital mucosa

**DOI:** 10.1038/s41598-019-38547-1

**Published:** 2019-02-13

**Authors:** María Paula Caruso, Juliana Falivene, María Pía Holgado, Diego Hernán Zurita, Natalia Laufer, Carina Castro, Ángeles Nico, Cynthia Maeto, Jimena Salido, Héctor Pérez, Horacio Salomón, Pedro Cahn, Omar Sued, Valeria Fink, Gabriela Turk, María Magdalena Gherardi

**Affiliations:** 1 Instituto de Investigaciones Biomédicas en Retrovirus y SIDA (INBIRS), Universidad de Buenos Aires-CONICET, Buenos Aires, Argentina; 2 Hospital J.A. Fernández, Buenos Aires, Argentina; 3 Fundación Huésped, Buenos Aires, Argentina; 4 Centro Medico Accord, Buenos Aires, Argentina

## Abstract

The aim of this study was to evaluate the effectiveness of antiretroviral treatment (ART) on the proportion and functions of Th17 and Treg cells in peripheral blood and female genital tract (FGT) respectively. To this aim, samples from 41 HIV-neg, 33 HIV+ ART-naïve and 32 HIV+ ART+ subjects were obtained. In peripheral blood, altered Th17 and Th17/Treg proportions were normalized in HIV+ ART+, but certain abnormal Treg and activated T-cell proportions were still observed. In FGT, abnormal patterns of secretion for Th17-related cytokines were observed in cervical mononuclear cells (CMCs) from HIV+ women, even in those from HIV+ ART+, compared to the HIV-neg group. Moreover, these altered patterns of secretion were associated with diminished levels of CXCL5 and CXCL1 chemokines and with an immunoregulatory skew in the CCL17/CCL20 ratio in ectocervix samples of these women. Finally, ART did not restore proportions of Th17-precursor cells with gut-homing potential in PBMCs, and positive correlations between these cells and the levels of IL-17F and IL-21 production by CMCs may suggest that a better homing of these cells to the intestine could also imply a better restoration of these cells in the female genital tract. These results indicate that antiretroviral treatment did not restore Th17-related immune functions completely at the female mucosal level.

## Introduction

Treg and Th17 cell subsets are characterized by the expression of specific transcriptional factors, chemokine receptors and by the secretion of specific cytokine and chemokines. These subsets are important for the differentiation, expansion, homing capacity, and recruitment of several different immune cell populations to the site of infection^1^. Notably, both T cell subsets play crucial roles in mucosal tissues by maintaining the mucosal barrier integrity (Th17 cells) and preventing inflammation (Treg cells)^2^. Th17 cells a CD4^+^ T-cell subset of a lineage different from Th1 and Th2, is characterized by the secretion of a distinctive pattern of cytokines: IL-17A, IL-17F, IL-21 and IL-22, involved in the *in vivo* function of these cells^3,4^. Th17 cells play an essential role in mucosal immunity, maintaining thus the mucosal barriers^5,6^, and working in the response to extracellular bacteria and fungi by promoting neutrophil recruitment^7,8^, or by inducing epithelial cells to produce antimicrobial peptides such as β-defensin 2 (hBD-2) and hBD-3^9^, and mucins such as MUC5AC and MUC5B^10^. Regulatory T cells constitute a specialized subpopulation of CD4+ T lymphocytes that are critical to the immune balance and to the effective functioning of the immune system, both in normal and diseased states. Treg cells mediate their suppressive function by controlling the activation and expansion of immune cells. They control inflammation by producing immunosuppressive cytokines^11^ and inducing cytokine deprivation apoptosis of effector CD4+ T cells^12^. The functional effect of Tregs on HIV immune pathogenesis remains poorly understood. Thus, while some findings have revealed a beneficial effect through the suppression of chronic immune activation, others observe a detrimental role since the inhibition of specific HIV immune response through suppressive potential can promote viral persistence in the host^13,14^. Different works have demonstrated that SIV and HIV infections lead to selective depletion of Th17 cells in both blood and gastrointestinal lymphoid tissues that can predict disease progression^15,16^. Indeed, many studies highlight the importance of the Th17/Treg ratio in disease progression during HIV-1 and SIV infections^1,17^. Our previous study described the relevance of Th17 cells during primary HIV infection (PHI)^18^, finding an association between a better clinical status with higher Th17 and lower Treg levels. Most important, for the first time we demonstrated that during PHI, higher Th17 levels directly correlated with more potent HIV antiviral T-cell responses associated with protection.

The events that occur at the genital mucosa level play a prominent role in HIV immunopathogenesis, as it is the place where the initial viral replication occurs after vaginal transmission of HIV in women and SIV in macaques^19,20^. In relation to the relevance of Th17 cells in the mucosal genital tract during HIV infection, a pronounced depletion of this T-cell subset was described in the cervical mucosa from HIV+ female sex workers compared to HIV-neg women^21^. Another study from the same authors showed that a reduction in the frequency of Th17 cells in the cervical mucosa takes place during early HIV infection^22^, suggesting a similar scenario to that found in the intestine. Even more, in the SIV model Stieh *et al*., corroborated these previous findings identifying Th17 lineage CCR6+ CD4+ T-cells as primary targets for SIV during vaginal transmission^23^. One of the answers to the faster decrease in this T-cell subset, could be related to a higher susceptibility of Th17 cells to HIV-infection^24,25^, in line with their earlier disappearance from the gut and cervix of HIV-infected patients.

Due to the relevance of Th17 cells and Th17/Treg balance during HIV infection, the analysis of the restoration of these parameters upon antiretroviral treatment (ART) is necessary. Thus, several studies have analyzed the effects of ART on the restoration of Th17 cells focused on the analysis of PBMCs and the intestinal mucosa. Results indicated that in many cases, although viral load was suppressed, Th17 responses were not restored in all the patients^26,27^. On the other hand, recent studies have demonstrated that the intestinal mucosal Th17 cell function is altered during HIV infection and although ART could normalize Th17 cell numbers, functionality restoration was delayed^28^. Even when ART is initiated at early stages post-infection, such as Fiebig III stage, Th17 cell polyfunctionality could not be restored^29^. Although HIV infection causes depletion of CD4+ Tregs leading to their lower absolute cell numbers in blood and gut mucosa^30^, FOXP3+ Tregs are observed in increased proportions in relation to Th17 cells in gut mucosa and oral mucosa during SIV/HIV infection^17,31,32^. In this sense, it has been proposed that pro-inflammatory milieu in ART-treated patients with immune activation significantly contributes to an enhanced loss of Th17 cells and an increased in the frequency of dysregulated Tregs in the mucosa, which in turn may exacerbate immune dysfunction in HIV-infected patients^2^.

It is known that during pregnancy, the maternal immune system needs to adapt to tolerate a semi-allogeneic fetus, both Treg cells and uterine NK cells appear to participate in this process^33^. However, there is little information about whether Treg cells are more abundant in the reproductive tract of HIV-infected individuals as compared to healthy controls, or whether Treg cells modulate T cell responses and/or immune activation in reproductive tissues^34^. In this regard, it has been described that HIV-1 highly-exposed seronegative (HESN) female commercial sex workers (CSWs) had higher frequencies of endocervical regulatory CD4^+^ T-cells when compared to both HIV-1-infected CSWs and HIV-1-uninfected non-CSWs^35^.

On the other hand, although it has been described that Th17 cells are highly enriched in the mucosal female reproductive tract^36^ and that after HIV infection a significant depletion of this T-cell subset in this mucosal compartment occurs^21,22^, until now there is no study in which they have evaluated in detail Th17-cell functionality or reestablishment in FGT after ART initiation. The only data available comes from the work by Masson *et al*.^37^, in which the authors described that cervical Th17 cell frequencies were similar in HIV-uninfected and HIV-infected women (ART+ or ART naïve).

Thus, taking into account the disturbance that occurs with Th17 and Treg cells during HIV infection and the important role played by both T-cell subsets have in the mucosal immunity, the aim of this study was to evaluate the recovery in terms of quantity and functionality of the before-mentioned subpopulations in periphery and in the female genital tract after the administration of antiretroviral treatment.

## Results

### ART restores Th17 proportions and Th17/Treg ratios in blood, but abnormal T-cell activation and Treg levels were still maintained

In our previous report^18^, Th17 and Treg subsets and their correlation with HIV-specific T-cell responses and clinical parameters were analyzed during (acute/early) primary HIV infection (PHI). We found that, percentages of Th17 and Treg subsets were severely altered in ART-naïve chronically-infected individuals, whereas an imbalance of Th17/Treg ratio was observed in all HIV+ groups compared to healthy donors (HDs), including PHI at baseline and Elite controllers.

**Figure 1:**
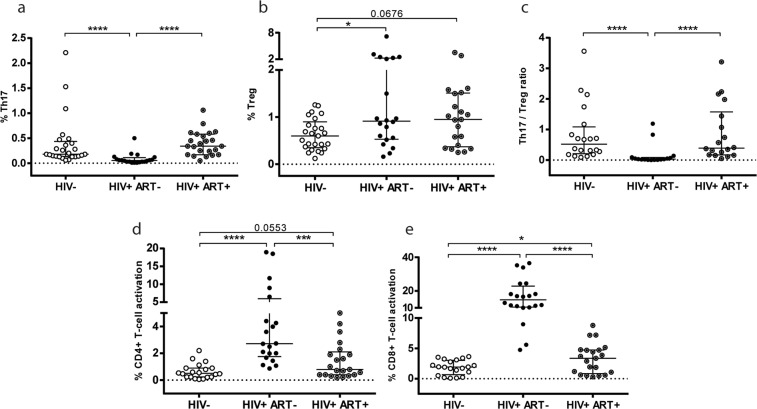
ART treatment restore Th17 proportions and Th17/Treg ratios in blood, however abnormal T-cell activation and Treg levels were still maintained. Proportions of Th17 and Treg cells were determined as described in Materials and Methods. (**a**) Values of CD3+ CD4+ IL-17+ producing cells (%Th17) are shown. (**b**) CD3+ CD4+ CD25+ FoxP3+ cells (%Treg). (**c**) Th17/Treg ratio was calculated for each patient. (**d**) Proportions of CD4+ T-cell activated cells (%CD3+ CD4+ CD38+ HLA-DR+) and (**e**) CD8+ T-cell activated cells (%CD3+ CD8+ CD38+ HLA-DR+) are shown. Lines indicate median and interquartile range (IQR). Symbols represent individual patients: HIV-neg (○), HIV+ ART− (●), HIV+ ART+ (◉). The *p* values obtained are depicted as *p < 0.05, **p < 0.01, ***p < 0.001 and ****p < 0.0001.

Thus, taking into account these important effects that occurred during HIV infection, our first inquiry was to evaluate the ability of ART to normalize these parameters. As the majority of HIV+ individuals diagnosed in Argentina initiate ART during the chronic phase of infection, we considered important to analyze treatment effectiveness in these patients. Therefore, the HIV+ subjects on treatment recruited in the study had all initiated ART during the chronic phase of infection with a median (IQR) of 6.5 (3.00–15.50) years on treatment and a median of 3.33 (1.48–5.25) years with documented suppressed VL (Table 1). Additional data specifying the drugs applied in the ARTs is described in (Supplementary Table S1). Figure 1(a–c) shows that in the group of chronically-infected patients still in absence of treatment (HIV+ ART−), percentages of Th17, Treg cells, and Th17/Treg ratios were significantly altered compared to the values observed in the HIV-neg group. Thus, proportions of Th17 and Treg cells found in HIV+ ART− were approximately 1/3 fold less and 1,5 fold higher respectively, in relation to the levels found in the HIV-neg group. In consequence, the alterations in both T-cell subpopulations (lower Th17 and higher Treg proportions) produced significantly lower Th17/Treg ratios in HIV+ ART− [0.03 (0.0125–0.085) *vs*. HIV-neg 0.52 (0.2050–1.085); p < 0.0001)]. On the other hand, in the HIV+ ART+ group we found that the median (IQR) of %Th17 and Th17/Treg ratio values (Fig. 1a,c) were restored [Th17: 0.34 (0.17–0.58), Th17/Treg ratio: 0.39 (0.1675–1.575)]. Significant differences were found compared to the group without treatment (p < 0.0001); while in the HIV-neg group, differences were not significant (Th17: p = 0.1160 and Th17/Treg ratio: p = 0.9367).

**Table 1:** Clinical characteristics of groups of individuals enrolled for the study.

Group	HIV− (n = 41)	HIV+ ART− (n = 33)	HIV+ ART+ (n = 32)
Age (years) median (IQR)	34 (27–42)	35 (28–43)	41 (35–47)
Sex (%females)	75	60	100
Time after first positive serology median (IQR) (years)	—	2.00 (0.42–9.00) (n = 11) or ND (n = 22)	14.00 (6.75–19.00)
Time under ART median (IQR) (years)	—	—	6.50 (3.00–15.50)
Time under ART with documented suppressed VL^a^ median (IQR) (years)	—	—	3.33 (1.48–5.25)
Log_10_ VL^a^ mean ± SD	—	4.53 ± 0.95	<1.70
CD4 counts^b^ median no. of cells/ul (IQR)	810 (569–989)	344 (85–568)****	564 (432–676)**
CD8 counts^b^ median no. of cells/ul (IQR)	493 (358–665)	771 (538–1209)**	739 (597–921)***
CD4/CD8 ratio median (IQR)	1.62 (1.36–1.92)	0.36 (0.06–0.56)****	0.80 (0.53–1.02)****^, ++^
%CD4 median (IQR)	43.00 (40.73–46.50)	21.38 (10.50–34.5)****	31.30 (25.18–37.09)****^, ++++^

ART: antiretroviral treatment. IQR: inter quartile range. ND: not determined. VL: viral load. SD: standard deviation. ^a^Versant HIV-1 RNA 3,0 assay, Siemens. Lower and upper detection limits are 40 and 500.000 RNA copies/mL, respectively (1.7 and 5.7 log). VL supressed: <40 copies/mL. ^b^CD4 and CD8 counts were determined by flow cytometry double platform, FACSCanto, BD, Bioscience. *p < 0.05, **p < 0.01, ***p < 0.001, ****p < 0.0001 comparisons to HIV− group. ^++^p < 0.01, ^++++^p < 0.0001 differences between HIV+ ART− and HIV+ ART+ groups.

When percentages of Treg cells were evaluated (Fig. 1b), the values found in HIV+ ART+ were still similar to those detected in patients without treatment [0.95% (0.37–1.51) HIV+ ART+ *vs*. 0.915% (0.53–2.26) HIV+ ART−; p = 0.8], while values tended to be higher compared to the HIV-neg group [0.95% (0.37–1.51) HIV+ ART+ *vs*. 0.6% (0.35–0.9) HIV−; p = 0.0676].

Immune activation constitutes a hallmark of HIV infection, and was documented to occur at early times post-infection^38,39^. Moreover, in some cases, the antiretroviral treatment failed to restore T-cell activation status to levels found in HIV− persons, as reported elsewhere^29,40,41^. CD4+ and CD8+ T-cell activation was evaluated by measuring the expression of the activation markers CD38 and human leukocyte antigen (HLA)–DR, on the surface of T cells. This analysis was performed in the different groups (Fig. 1d,e), and as expected, the highest proportions of CD4+ and CD8+ activated T-cells were detected in the HIV+ ART− group [2.72% (1.75–5.975) and 14.77% (10.42–22.84) *vs*. 0.5% (0.24–0.9) and 1.92% (0.66–3.03) in HIV-neg; p < 0.0001]. In fact, in this HIV+ group, T-cell activation levels were incremented nearly 5.4 (CD4+) and 7.7 (CD8+) fold compared to the baseline levels found in HIV-neg subjects. Importantly, after treatment, CD4+ and CD8+ T-cell activation values decreased being significantly lower regarding those detected in ART-naïve patients [in HIV+ ART+: CD4+: 0.8% (0.4–2.1) and CD8+: 3.37% (0.85–4.74); p < 0.0001]. However, when the T-cell activation magnitude in HIV+ ART+ patients was compared to those found in HIV-neg subjects, higher levels were still observed in the HIV+ ART+ group [in CD4+ p = 0.055 (Fig. 1d) and in CD8+ p = 0.0479 (Fig. 1e)]. In this case, the increase in T-cell activation was approximately 1.6 (CD4+) and 1.75 (CD8+) fold compared to the levels found in the HIV-neg group.

### T-cell activation magnitude in HIV+ ART+ correlated directly with proportion of total Treg-cells and indirectly with functional Treg (%Treg CD39) and Th17/Treg ratios

In the results described above, treatment was found to be effective in decreasing significant quantities of T-cell activation. However, the levels found in HIV+ ART+ were not as low as those detected in the HIV-negative group.

**Figure 2:**
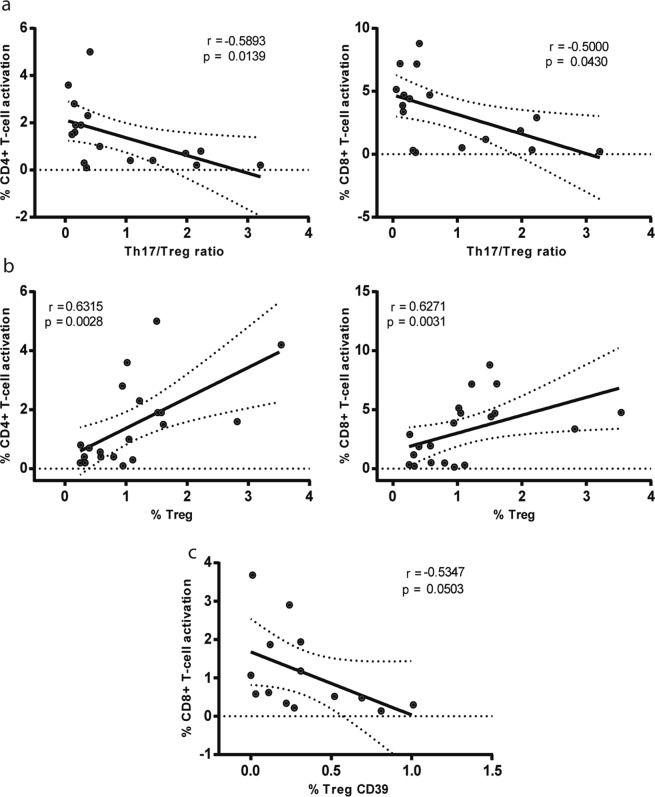
T-cell activation magnitudes in HIV+ ART+ were positively correlated with proportion of total Treg-cells and indirectly with functional Treg (%Treg CD39) and Th17/Treg levels. Correlations found among HIV+ ART+ patients. (**a**) Th17/Treg ratio versus (vs) %CD4+ T-cell activation (left panel) and vs %CD8+ T-cell activation (right panel); (**b**) %Treg vs %CD4+ T-cell activation (left panel) and vs %CD8+ T-cell activation (right panel) and (**c**) %Treg CD39+ vs %CD8+ T-cell activation. Symbols represent individual patients: HIV+ ART+ (◉). All *r* and *p* values correspond to Spearman’s correlations.

Later, in a more in-depth analysis of possible relationships between Th17 and Treg subpopulations and T-cell activation status, higher values of Th17/Treg ratio in the HIV+ ART+ group correlated with both lower T-cell activation levels of CD4+ and CD8+ cells (r = −0.589; p = 0.014 and r = −0.5; p = 0.043) (Fig. 2a). On the other hand, a positive correlation was observed between both CD4+ and CD8+ T-cell activation and %Treg cells (r = 0.631; p = 0.0028 and r = 0.627; p = 0.0031) Fig. 2b left and right panel.

CD39 has been described as a surface marker of functionally active human Treg cells^42^. Interestingly, when this marker was evaluated on CD4 Treg cells (%Treg-CD39+) in the HIV+ ART+ group (Fig. 2c), we found an inverse correlation was observed between the percentage of functionally active Treg cells and CD8+ T-cell activation levels (r = −0.535; p = 0.05). The same analysis was also performed in HIV-negative individuals, but in this case it was only possible to do it in six samples, and we did not found a significant correlation (r = 0.2; p = 0.7139).

### HIV infection significantly altered the production of Th17-related cytokines from cervical mononuclear cells of mucosal genital tract and ART could not restore this effect completely

The most relevant functions of Th17 and Treg cells are exerted in the mucosal compartments, thus the evaluation of their reconstitution or functionality at the mucosal level after ART in HIV infection is mandatory. Although different studies analyzed this issue at the mucosal intestinal compartment level, less data has been reported to date in relation to the female mucosal genital tract.

Taking this into account, different female mucosal genital tract samples were obtained from women of the three study groups (as described in Materials and Methods) in order to perform different specific assays. Cervical cytobrush-derived cervical mononuclear cells (CMCs) obtained from the different groups, were activated *in vitro* with PMA/Ionomycin during 5 hours as described in Materials and Methods. Then, Cytokines were determined by CBA in cell-culture supernatants, as follows: Th17-related pattern: (IL-17A, IL-17F, IL-21 and IL-22), and Treg-pattern: (IL-10 and TFG-β1). In a qualitative analysis (see Supplementary Fig. S2), the HIV+ ART− group showed a clearly diminished proportion of positive responses for Th17-related Cytokines (IL-17A, IL-17F and IL-21) in relation to the HIV-neg subjects. In the HIV+ ART+ group, significant lower proportions of positive responses compared to the HIV-neg group were still found for IL-17A and IL-17F. In the case of Treg-related Cytokines (IL-10 and TGF-β1) (Fig. S2a, right panel), significant reductions were only found in the proportion of IL-10 positive responses. The analysis of the number of Cytokines secreted by CMCs (Fig. S2b), showed significant differences in the Th17-related cytokines, when comparing HIV+ groups with the HIV-neg group, whereas the analysis of the number of Treg-related cytokines secreted (IL-10 and TGF-b) (Fig. S2b right panel) revealed no significant differences.

**Figure 3:**
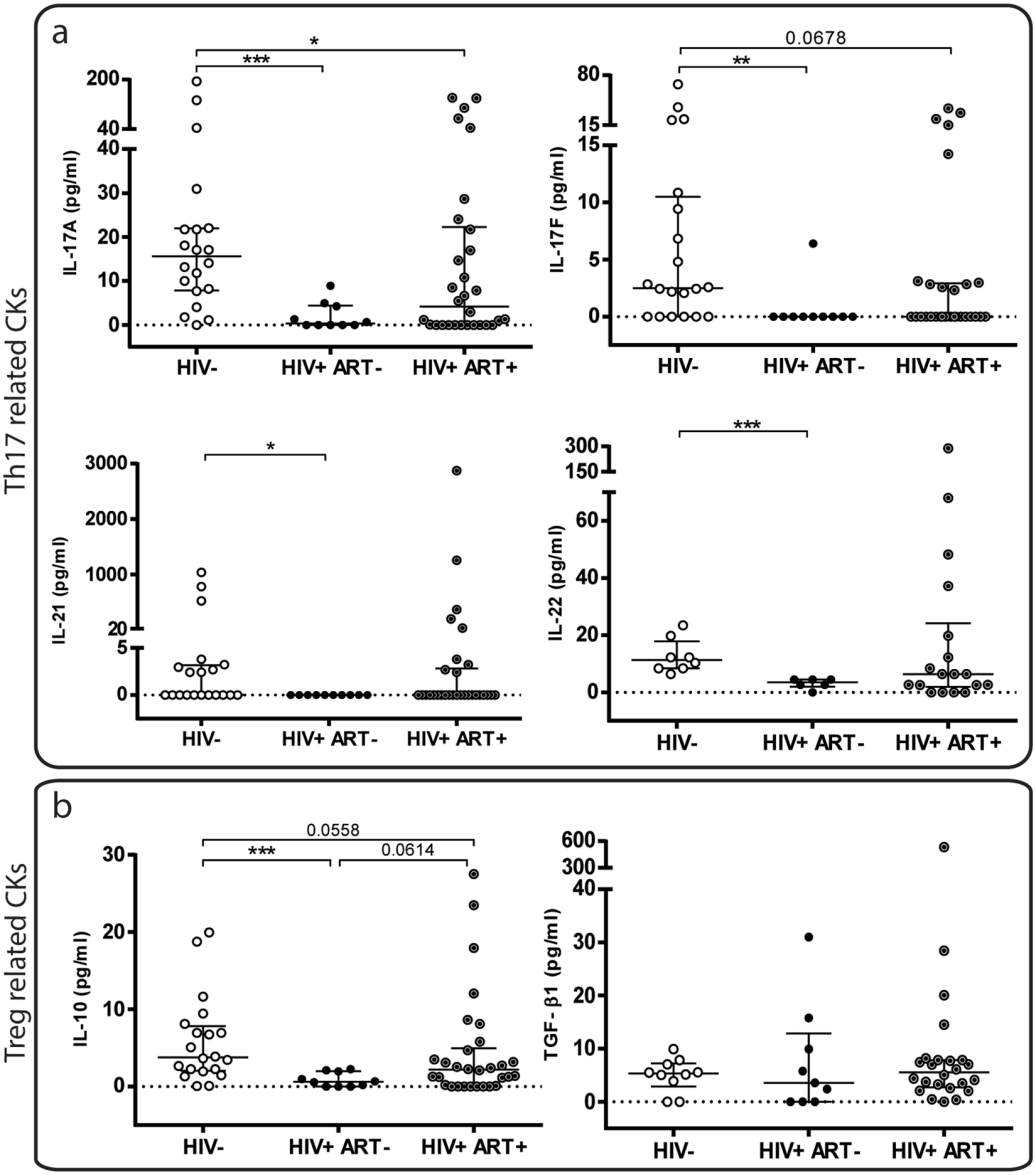
Quantitative analysis of the Th17 and Treg-related cytokines (cks) production from stimulated Cervix Mononuclear Cells (CMCs). (**a**) Levels (pg/ml) of IL-17A, IL-17F, IL-21, IL-22 (Th17 related cks) and (**b**) IL-10 and TGF-β1 (Treg-related cks) detected in the CMCs supernatants after PMA/ionomycin activation are shown. Lines indicate median and IQR. Symbols represent individual patients: HIV-neg (○), HIV+ ART− (●), HIV+ ART+ (◉). (**b**) The *p* values obtained are depicted as *p < 0.05, **p < 0.01 and ***p < 0.001.

Then a quantitative analysis of CMCs secreted cytokines (Fig. 3), revealed that in the absence of treatment (HIV+ ART−), the levels of the four Th17-Cytokines (Fig. 3a) and of IL-10 (Fig. 3b) were significantly lower compared to those observed in the HIV-neg were detected. After treatment, larger amounts of Cytokines were generally observed, although lower levels were still found compared to the values found in the HIV-neg group. Thus, the median IL-17A values observed in HIV-neg women were 15.58 pg/ml (7.83–21.97) in contrast to 0.34 pg/ml (0–4.418) found in HIV+ ART− (p < 0.01) (47-fold reduction) and 4.185 (0–22.31) in HIV+ ART+ (p < 0.05) (3.7-fold reduction). In the case of IL-17F, the median values found in the HIV-neg group were 2.51 pg/ml (0–10.5) *vs*. 0 (0–0) in HIV+ ART− (p < 0.01) and 0 (0–2.9) in HIV+ ART+ (p = 0.0678). Figure 3b shows the levels of Treg-related cytokines (IL-10 and TFG-β1) detected and it can be seen that for TGF- β1 similar median values were detected in the samples of the three groups (p > 0.05) (Fig. 3b right panel). Whereas for IL-10, significant lower quantities were found in HIV+ ART− compared to HIV-neg: [0.62 (0–1.995) *vs*. 3.76 (2.04–7.81); p < 0.001 (6-fold reduction)]. In samples from HIV+ ART+, IL-10 median values were incremented but still remained somehow lower compared to HIV-neg (2.175 (0.06–4.968) (p = 0.0558) (1.72-fold reduction).

### Analysis of the secretion pattern of Th17 and Treg cytokines indicated that HIV infection significantly altered functionality of genital mucosal CMCs for Th17-related cytokines

**Figure 4:**
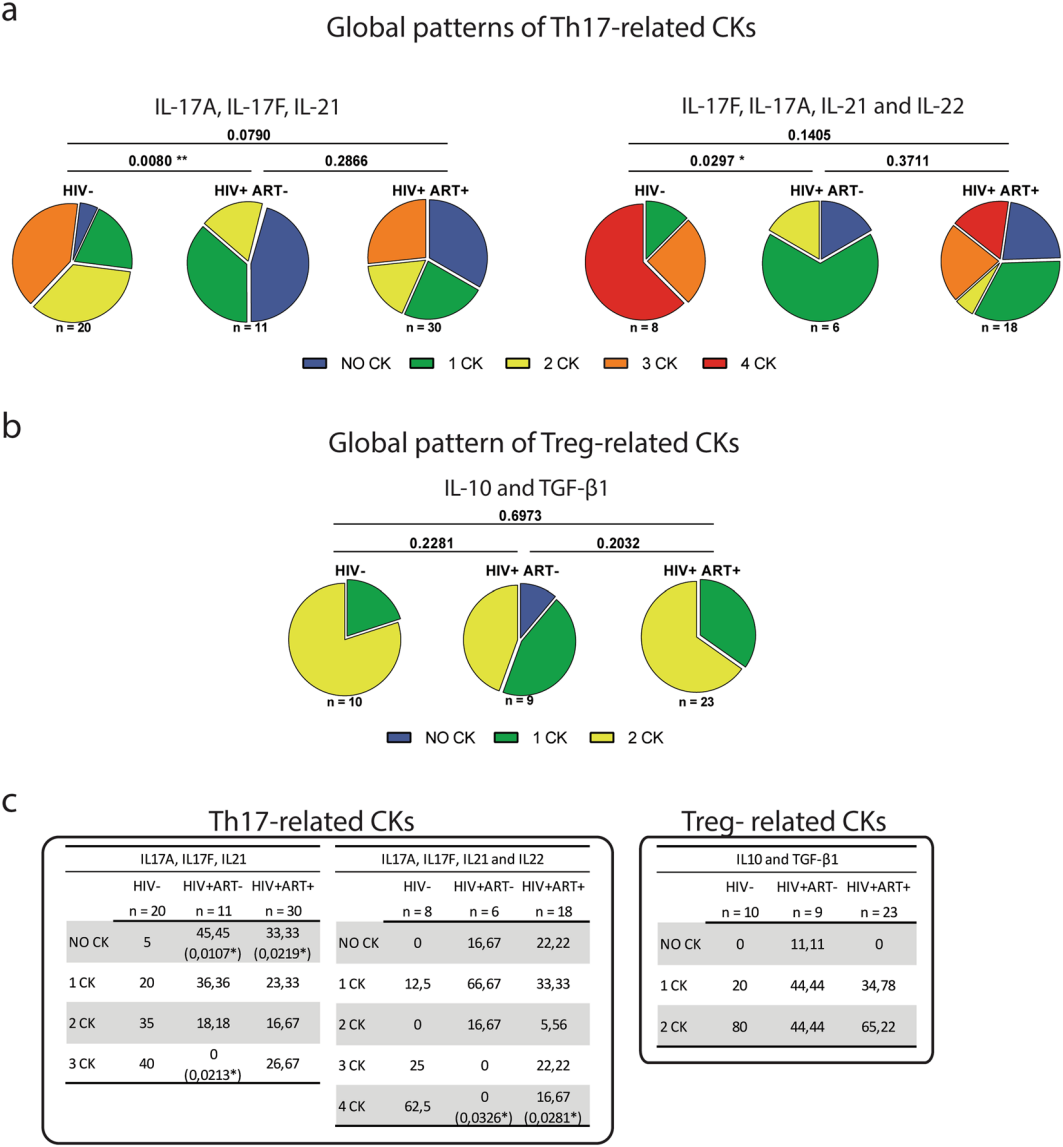
Secretion patterns of Th17 and Treg-cks indicated that HIV infection altered functionality of genital mucosal CMCs for Th17-related cytokines. (**a**) Global Th17-related ck patterns detected in the CMCs samples from the different groups. IL-17A, IL-17F, IL-21 and IL-22 production was evaluated in the supernatants by CBA, IL-22 production was determined depending on sample availability. Pie-charts represent the percentage of samples in each group with no ck, 1ck, 2cks, 3cks, or 4 cks detected for each of the Th17 global patterns represented. (**b**) Global Treg-related ck patterns detected in the CMCs samples from the different groups. IL-10 production was evaluated in the supernatants by CBA and TGF-β1 was evaluated by ELISA depending on sample availability. Pie-charts represent the percentage of samples in each group with no ck, 1 ck or 2 cks detected for the Treg-related cytokine global pattern. Significant differences between global patterns are indicated. Different colors represent different number of cytokines produced, as indicated in the figure. Below each pie it is indicated the number of subjects in which the global pattern was evaluated. (**c**) Tables show the percentages of samples in each of the patient groups with the indicated number of cytokines present in the CMCs samples. Left panel: Th17-related cks, right panel Treg-related cks. The *p* values shown are in comparison to the HIV-neg group and are depicted as *p < 0.05 and **p < 0.01.

A more in-depth analysis was conducted aimed at evaluating whether the infection with HIV could produce an alteration in the pattern of Th17 and Treg-related cytokines secreted by CMCs. This analysis was done as an indirect measure of the functionality of these cells at the mucosal genital tract level. To this, we constructed the Th17 and Treg-cytokine global patterns detected in the supernatants of the CMCs samples of the different groups and statistical comparisons were performed by Chi square test (Fig. 4a,b). Thus, for the HIV-neg group when IL-17A, IL-17F and IL-21 were measured for the global Th17 pattern (Fig. 4a left panel), this consisted in a proportion of 40%, 35%, 20% and 5% of samples with 3 cytokines, 2 cytokines, 1cytokine or none-cytokine respectively. For the HIV+ ART− group this global pattern of secretion was severely modified, as in this case, the proportion of samples with 3 cytokines, 2 cytokines, 1 cytokine or none-cytokine were: 0%, 18.18%, 36.36% and 45.45% respectively (in comparison with the HIV-neg pattern p < 0.01). A similar scenario was observed when four Th17-related cytokines (IL-17A, IL-17F, IL-21 and IL-22) were determined for the construction of the global Th17 pattern (Fig. 4a right panel), (HIV-neg vs HIV+ ART−: p < 0.05). In the HIV+ ART+ group, it can be appreciated that both global Th17-cytokine patterns (Fig. 4a) tended to be restored as significant differences in comparison with the HIV-neg group were not found. In contrast with the scenario observed in the Th17 patterns, no significant differences were found between the different groups in the Treg-cytokine global patterns (Fig. 4b), thus suggesting that during HIV infection even in the absence of treatment, functionality of CMCs in relation to IL-10 and TGF-β1 was not significantly altered.

Figure 4c depicts the percentages of CMCs samples with no-cytokine, 1 cytokine, 2 cytokine, 3 cytokine or 4 cytokine for Th17 (left panel) or no-cytokine, 1 cytokine or 2 cytokine for Treg (right panel) related cytokines of the three groups. Mirroring the scenario observed after the analysis of the global cytokine patterns described above, significant differences were only observed for the Th17-cytokines. Thus, whereas only 5% of the HIV-neg samples showed no cytokines (for IL-17A, IL-17F and IL-21) (Fig. 4c left panel), this proportion was incremented in 45.45% (p = 0.01) in HIV+ ART− samples and 33.33% (p = 0.022) in HIV+ ART+. On the other hand, we found 40% of samples with 3 cytokines in HIV-neg, in contrast with 0% of the HIV+ ART− samples with 3 cytokines (p = 0.021). After treatment (HIV+ ART+), this proportion was incremented in 26.67%, (p = 0.327) although not reaching the proportions observed in HIV-neg. When four Th17-related cytokines were analyzed (IL-17A, IL-17F, IL-21 and IL-22) (Fig. 4c right panel), the proportion of samples with 4 cytokines detected in HIV-neg was 62.5% in contrast to 0% (p = 0.0326) in HIV+ ART− and 16.67% (p = 0.0281) in HIV+ ART+.

Conversely, the analysis of the percentage of samples with no-cytokine, 1cytokine or 2cytokines for Treg-related cytokines (Fig. 4c right panel), showed none significant differences between groups for any case.

These last analyses indicated that in the absence of the ART global pattern of secretion of Th17-related cytokines of CMCs was severely altered, after treatment this global pattern tended to be restored. However, significant differences were still found in the proportion of samples with no and four Cytokines compared to the HIV-neg group.

### CMCs functionality (related to Th17 and Treg-cytokines) was associated with local mucosal genital chemokine levels

The results described above suggest that HIV infection severely affected the functionality of the female mucosal genital tract cells (CMCs), and the administration of antiretroviral treatment even after a relatively long period of time (see Table 1) was not able to restore the functional characteristics as observed in the absence of HIV infection (HIV-neg group). On the other hand, different studies have shown that HIV infection perturbed the local genital mucosal environment generating alterations in the baseline chemokine/cytokine levels^43,44^. Thus, our next aim was focused on the study of possible alterations in the chemokine levels of a total of 13 human pro-inflammatory chemokines in ectocervix samples from the different groups. Then, we analyzed whether CMCs functionality (in terms of Th17 and Treg-related cytokines) could have any relationship with the chemokines that were altered. Supplementary Table S3 describes the quantities of the specified chemokines detected in the supernatants of the ectocervix swabs from the different groups. Of note, when the total protein content was evaluated in the ectocervix samples, no significant differences were detected between the groups (data not shown), suggesting that a homogeneous quantity of material was obtained from all samples independently of the subject group.

**Figure 5:**
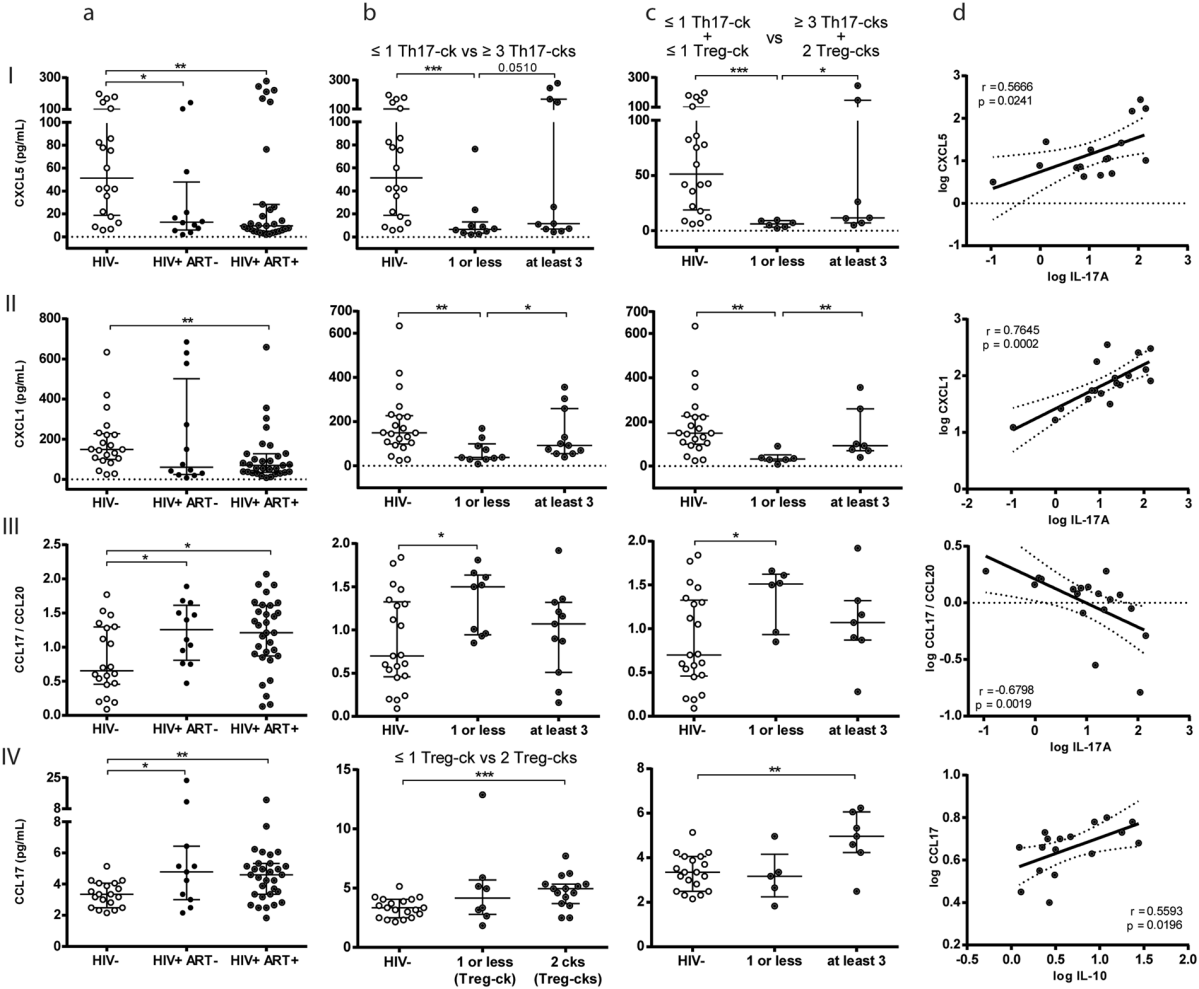
Functionality of CMCs (related to Th17 and Treg-cks) was associated with local mucosal genital chemokine levels. Chemokine levels were quantified by CBA (pg/ml) in ectocervix samples from the different groups as described in methodology (Column **a**): Row I: CXCL5 (ENA-78), (II) CXCL1 (GRO-α), (III) CCL17/CCL20 ratio (TARC/MIP-3α ratio) and (IV) CCL17 (TARC) quantities are shown. (Column **b** rows I to III): HIV+ ART+ group was subdivided in two sub-groups according to have “at least three” or “one or less” Th17-cks (≥3Th17-cks vs ≤1 Th17-ck) in the CMCs samples. (Column **b** row IV): HIV+ ART+ group was subdivided in two subgroups according to have two Treg- cks vs one or less Treg-ck (2 Treg-cks vs ≤1 Treg-ck). CXCL5, CXCL1, CCL17/CCL20 ratio and CCL17 levels are shown for HIV-neg and HIV+ ART+ sub-divided groups. (Column **c** rows I to IV): HIV+ ART+ group was subdivided in two subgroups according to have at least three Th17-cks plus two Treg-cks vs one or less Th17-cks plus one or less Treg-cks (≥3Th17-cks + 2Treg-cks vs ≤1 Th17-ck + ≤1 Treg-ck). CXCL5, CXCL1, CCL17/CCL20 ratio and CCL17 levels are shown for HIV-neg and HIV+ ART+ sub-divided groups. (Column **d**): Correlations found among HIV+ ART+ patients: (row I) log CXCL5 versus (vs) log IL-17A, (row II) log CXCL1 vs log IL-17A, (row III) log CCL17/CCL20 ratio vs log IL-17A and (row IV) log CCL17 vs log IL-10. Lines indicate median and IQR. Symbols represent individual patients: HIV-neg (○), HIV+ ART− (●), HIV+ ART+ (◉). The *p* values obtained are depicted as *p < 0.05, **p < 0.01 and ***p < 0.001. (Column **d**) *r* and *p* values correspond to Spearman’s or Pearson correlations.

Of the thirteen molecules evaluated, significant differences were detected in the HIV+ groups compared to HIV-neg for three of them (CXCL5, CXCL1 and CCL17). Remarkably, no significant differences were observed between the HIV+ groups (ART− *vs*. ART+), for none of the chemokines evaluated (Table S3). Figure 5 A depicts the levels of CXCL5, CXCL1 and CCL17 chemokines detected in the ectocervix samples of the different groups. The CXC chemokines: CXCL5 or ENA-78, and CXCL1 or GRO-α (Fig. 5 column a rows I and II) both use the same cellular receptor (CXCR2) and are capable of trafficking neutrophils as the main function. Notably, both chemokines were found in lower levels in both HIV+ groups in relation to the group of HIV-negative women. Thus, whereas median CXCL5 levels found in HIV-neg were 51.34 pg/ml (18.76–100.1), the levels detected in HIV+ ART− and HIV+ ART+ were 12.89 pg/ml (5.975–47.96) (p < 0.05) and 9.6 pg/ml (4.96–28.31) (p < 0.01). And for CXCL1, in HIV-neg, the median values were 149 pg/ml (98.87–226.9), *vs*. 69.87 pg/ml (34.66–127.1) in HIV+ ART+ patients (p < 0.01) and 60.28 pg/ml in HIV+ ART− (24.52–501.2) (p > 0.05, probably not significant due to a high variability of data). Another chemokine which showed significant differences between groups was CCL17 also known as TARC (thymus- and activation-regulated chemokine), involved in the homing of Treg cells. As observed in Fig. 5a,IV, the median CCL17 values found in HIV-neg were 3.35 pg/ml (2.49–4.06), while the values detected in samples from HIV+ ART− and HIV+ ART+ were significantly higher: 4.78 (3–6.43) (p < 0.05) and 4.6 (3.35–5.33) (p < 0.01) respectively.

CCL20 (MIP-3α), a chemokine important for the recruitment of Th17 cells, and also with functions in B cell and DC homing to gut-associated lymphoid tissue^45^, was also measured (see Supplementary Table S3) and although median values were slightly higher in HIV− *vs*. HIV+, differences between groups were not significant. But, when the CCL17/CCL20 (chemokine for Treg-cells/chemokine for Th17-cells) ratio was calculated (Fig. 5a,III), significant differences were found between groups, finding higher median CCL17/CCL20 ratio values for the HIV+ groups when compared to HIV− (p < 0.05).

**Figure 6:**
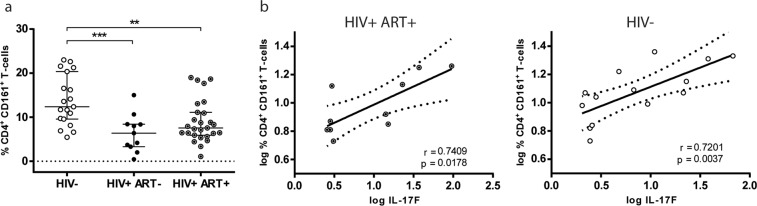
CD161+ CD4+ T-cells in peripheral blood were significantly reduced in HIV+ groups and were directly correlated with the genital mucosal production of IL-17F. (**a**) Proportions of CD4+ CD161+ T-cells in PBMCs of the different groups. Lines indicate median and IQR. The *p* values obtained are depicted as *p < 0.05, **p < 0.01, ***p < 0.001 and ****p < 0.0001. (**b**) Correlation of log %CD161+ CD4+ T-cells in PBMCs vs log IL-17F from CMCs within HIV+ ART+ (left panel) and HIV-neg (right panel) women. *r* and *p* values correspond to Spearman’s or Pearson correlations. Symbols represent individual subjects: HIV-neg (○), HIV+ ART− (●), HIV+ ART+ (◉).

Taking into account the alteration detected in the CMCs functionality, mainly for Th17-related cytokines, as described in the previous section, and that one of the effector functions of Th17-cytokines (ie: IL-17A and IL-17F) is the induction of CXC chemokines, our next aim was to inquire if that function deterioration could be associated with the chemokines with altered levels in the HIV+ groups. To achieve this, the HIV+ ART+ group was subdivided into two, depending on whether they had “at least three” or “one or less” Th17-cytokines (≥3Th17-cytokines *vs*. ≤1Th17-cytokine) in the CMCs samples. Then, the quantities of these chemokines were compared in these two subgroups and in the HIV-neg group (Fig. 5b,I–III). We also considered the Treg-cytokines secretion and thus in Fig. 5c the HIV+ ART+ group was subdivided into two depending on whether they had at least three Th17-cytokines plus two Treg-cytokines, or one or less Th17-cytokines plus one or less Treg-cytokines (≥3Th17-cytokines + 2Treg-cytokines *vs*. ≤1Th17-cytokine + ≤1Treg cytokine). Of note, for CXCL5 and CXCL1, when the group of HIV+ ART+ was subdivided, only significant low levels of chemokines were detected for the sub-group in which one or less Th17-cytokines were detected (p < 0.001 for CXCL5 and p < 0.01 for CXCL1), (Figs 5b,I and 6b,II). On the other hand, the subgroup with at least 3 Th17 cytokines showed levels of CXCL5 and CXCL1 higher than those detected in the subgroup of ≤1 Th17 cytokine (p < 0.05) and significant differences were not found any more compared to the HIV-neg group (Figs 5b,I and 6b,II). Similar, or even more significant results were found when the HIV+ ART+ group was subdivided if they had: ≥3Th17-cytokines + 2Treg-cytokines *vs*. ≤1 Th17-cytokine + ≤1 Treg-cytokine (Fig. 5c,I and II) in CMCs samples. Thus, the subgroup of HIV+ ART+ samples with ≥3Th17-cytokines + 2Treg-cytokines (more similar to those detected in HIV-neg samples) showed significant higher levels of CXCL5 and CXCL1 (p < 0.05 and p < 0.001) compared to those detected in the sub-group ≤1 Th17- cytokine + ≤1 Treg-cytokine. In line with these last results, significant direct correlations were found between the levels of CXCL5 or CXCL1 in ectocervix samples and IL-17A in supernatants from CMCs for the HIV+ ART+ group ((for CXCL5: r = 0.566, p = 0.02 (p = 0.07 after FDR adjustment); for CXCL1: r = 0.76, p = 0.0002 (p = 0.0012 after FDR adjustment)) (Fig. 5d,I and II respectively).

As already mentioned, higher values of the CCL17/CCL20 ratio (Treg chemokine/Th17 chemokine) were found in the HIV+ groups (Fig. 5a,III), and in this case when the HIV+ ART+ was subdivided, higher ratio levels were detected in the subgroup of patients with ≤1 Th17 cytokine (median 1.5 (0.94–1.63; p < 0.05 *vs*. HIV−), while significant differences *vs*. HIV-neg were lost when compared to the subgroup with ≥3Th17cytokines (Fig. 5b,III). Similar results were found for the CCL17/CCL20 ratio when the HIV+ ART+ group was subdivided accordingly if they had: ≥3Th17cytokines + 2Treg cytokines *vs*. ≤1 Th17-cytokine + ≤1 Treg-cytokine (Fig. 5c,III). Thus, significant differences compared to the HIV-neg group were only maintained with the group: ≤1 Th17-cytokine + ≤1 Treg- cytokine. Figure 5d,III shows that CCL17/CCL20 ratios in HIV+ ART+ patients indirectly correlated with IL-17A levels in supernatants from CMCs (r = −0.6798 p = 0.0019 (p = 0.011 after FDR adjustment)).

CCL17 was elevated in both HIV+ groups (Fig. 5a,IV). As this chemokine is involved in Treg chemotaxis, in this specific case the HIV+ ART+ group was subdivided accordingly to if there were two Treg-cytokines vs. one or less Treg-cytokines (2 Treg-cytokines vs. ≤1 Treg-cytokine) (Fig. 5b,IV). After this subdivision, significant higher levels of this chemokine were only detected in the group with 2 Treg-cytokines (Fig. 5b,IV), and when the analysis was performed according to the other criterion (≥ 3Th17Cytokines + 2Treg cytokines *vs*. ≤1 Th17-cytokine + ≤1 Treg-cytokine), higher values were still found in the group with 2 Treg cytokines (≥3Th17-cytokines + 2Treg-cytokines) (Fig. 5c,IV). For this chemokine, a significant positive correlation with IL-10 levels was observed in supernatants from CMCs (Fig. 5d,IV) (r = 0.56; p = 0.0196).

### Proportions of CD161+ CD4+ T-cells in peripheral blood were significantly reduced in both HIV+ groups (ART− and ART+) compared to HIV-neg, and were directly correlated with the genital CMCs production of IL-17F and IL-21

It has been described that Th17 cells arise from a population of CD161+ CD4+ T-cells and that this precursor population has gut-homing potential^46,47^. On the other hand, it has also been reported that HIV-infected individuals showed a reduced number of peripheral CD161+ CD4+ cells, which may limit Th17 restoration in mucosal compartments^16^. Taking into account this background information, we aimed to quantify this T-cell population in peripheral blood of the three study groups in order to evaluate the ability of the treatment to restore this Th17 precursor cells with mucosal gut-homing potential.

In Fig. 6a it can be seen that significantly minor proportions of CD161+ CD4+ T cells were detected in HIV+ subjects compared to HIV-neg, as the median percentages found in HIV-neg were 12.37 (9.56–20.37) compared to the median percentages values of 6.37 (3.3–8.41) (p < 0.001) and 7.56 (5.91–11.10) (p < 0.01) detected in HIV+ ART− and HIV+ ART+ groups respectively. It is noteworthy, that in contrast with what it was observed when percentages of Th17 cells were evaluated by intracellular IL-17A staining (Fig. 1a), it seems that proportions of CD161+ CD4+ T-cells were not restored after ART.

Finally, a positive correlation between the proportions of CD4+ CD161+ T-cells in PBMCs and the levels of IL-17F production by CMCs from endocervical samples (Fig. 6b) was found. This positive correlation was observed in the HIV+ ART+ group (Fig. 6b left panel) (r = 0.741, p = 0.018; p = 0.07 after FDR adjustment) and in the HIV-neg group, even with stronger significance (r = 0.72, p = 0.0037; p = 0.014 after FDR adjustment) (Fig. 6b right panel). Even more, a similar positive correlation was also found between % CD4+ CD161+ T-cells and log IL-21 production from CMCs in both groups (HIV−: r = 0.826, p = 0.016; p = 0.o3 after FDR adjustment, HIV+ ART+: r = 0.786, p = 0.048; p = 0.09 after FDR adjustment) (see Supplementary Fig. S4). These last results indicate that the presence of higher proportions of this Th17 precursor cell-population with mucosal homing potential is directly associated with higher production levels of the Th17-related cytokines such as IL-17F and IL-21 by CMCs.

## Discussion

The relevance of both Th17 and Treg cells in HIV immunopathogenesis in addition to the crucial roles played by these T-cell populations in maintaining the mucosal barrier integrity and preventing inflammation, suggests a need for an in-depth analysis of restoration in HIV+ individuals on ART.

In this study, proportions of Th17 and Treg cell subsets and Th17/Treg values were analyzed in PBMCs from a group of patients with a median of 6.50 years on treatment, and a median of 3.33 years with corroborated VL suppression. Percentages of Th17 and Th17/Treg values were found to be restored, showing no significant differences compared to the HIV-neg group. However, proportions of Treg cells in the HIV+ ART+ group were still higher in relation to the proportions detected in HIV-neg. In a previous report performed with PBMCs^48^ the authors found that after a median of three years of treatment, the proportions of Th17 cells were restored, and the %Treg cells diminished to values comparable to those found in the HIV− group. However, in that study contrary to what has been reported in many reports, the Th17/Treg ratios found in HIV+ naïve subjects did not show significant differences with respect to HIV-neg. Whereas in another study, after 12- months treatment, although Th17/Treg values were reestablished, percentages of both Th17 and Treg did not even reach those values found in healthy donors^49^. When T-cell activation levels were evaluated in peripheral blood in the different study groups, the elevated T-cell activation proportions observed in naïve HIV+ subjects, were significantly reduced in HIV+ ART+ patients. However, the levels of both CD4+ and CD8+ T-cell activated cells detected in this latter group were somehow even elevated compared to HIV-neg individuals, in line with data from previous reports^29,38–41^. Interestingly, we found that when the proportion of CD39-Treg cells was analyzed in the HIV+ ART+ group, an inverse correlation between the percentage of functionally active Treg cells (FoxP3+ CD39+) and levels of CD8+ T-cell activation was detected. CD39 was described as a surface marker of functionally active human Treg cells^42^, and although there is still controversy regarding the role of CD39 in HIV infection, it can be hypothesized that CD39+ Tregs are likely critical for the down modulation of T-cell immune activation, reducing niches for HIV replication^50,51^.

Undoubtedly, an important issue addressed in this work was the analysis of the effects of ART on the functionality of mucosal cells from the female reproductive tract by studying the production of Th17-related and Treg-related Cytokines by CMCs after PMA plus ionomycin activation. Qualitative and quantitative analyses of the CMCs secreted cytokines revealed that in the absence of treatment (HIV+ ART−), significant reductions were found in the proportion of samples with positive responses for the Th17-related cytokines and also in the levels of the four Th17-cytokines and of IL-10, compared to values found in HIV-neg women. After treatment (HIV+ ART+), although higher quantities of cytokines were detected generally, a tendency to find lower levels of cytokines *vs*. the HIV-neg group was still observed.

Functionality of T-cells can be measured as the capacity to secrete multiple cytokines in order to exert their effector activities in an optimal manner. In this study, we generated the Th17 and Treg cytokine global patterns of secretion detected after PMA plus ionomycin stimulation of CMCs to evaluate the functionality of these cells in relation to the secretion of these cytokines (Fig. 3). Results showed that global patterns of secretion for Th17-related cytokines were severely affected in CMCs from both groups of HIV-infected women, whereas the pattern of secretion of Treg cytokines was not significantly altered. Previous reports have described impaired functionality of intestinal mucosal Th17 cells during HIV infection. Thus, for instance Kim *et al*.^28^, showed that polyfunctionality of intestinal Th17 cells was severely altered post-infection and that these functional alterations occur even earlier than the reductions in Th17 numbers, being less readily reversed after ART initiation. In another elegant study performed with 42 individuals with acute HIV infection (AHI) (Fiebig (F) stage I–V), the authors showed that ART initiated at FI/II stages prevented loss of Th17 cell numbers and functionality. However, late treatment initiation (at FIII stage) restored Th17 cell numbers but not their polyfunctionality^29^. Nevertheless, to our knowledge, there are no reports focused on the effects of ART on the functionality of female mucosal genital cells in relation to Th17 or Treg-related cytokines. Interestingly, in a recent study based on the characterization of the presence of CD161+ T cells in the female reproductive tract of HIV-neg women^52^, the authors demonstrated that the cervical compartment was significantly more polyfunctional than the blood compartment with both CD161+ and CD161− Th cell fractions harboring elevated proportion of cells secreting three cytokines (IL-17, IL-22 and IFN-g) simultaneously. However, the functionality of cervical CD161+ Th17 cells in HIV+ women was not analyzed.

Analysis of thirteen chemokines in ectocervix samples showed that two of them (CXCL5 and CXCL1) characterized to be neutrophil-chemoattractants, were detected in significant lower levels in both HIV+ groups compared to the levels found in HIV-neg women. To inquire if the alteration in the CMCs functionality, principally for Th17-related cytokines, could be associated with the altered chemokine levels observed, the HIV+ ART+ group was subdivided into two, when there were “at least three” or “one or less” Th17 cytokines in CMCs samples. After this subdivision, only significant lower levels of the chemokines were detected in the sub-group in which one or less Th17 cytokines were detected, whereas the subgroup with at least three Th17 cytokines showed no significant differences compared to the chemokine levels found in HIV-neg ectocervix samples. Of note, these differences were maintained or even enlarged when secretion of Treg-related cytokines was also considered for the subdivision of the HIV+ ART+ group (≥3Th17cytokines + 2Treg cytokines vs ≤1 Th17 cytokine + ≤1 Treg cytokine). These results may indicate that CMCs functionality in relation to the capacity to secrete multiple Th17 cytokines is associated with the quantities of CXCL5 and CXCL1 chemokines present in ectocervix. In agreement with the fact that one of the main functions of Th17 cells is the recruitment of neutrophils, thus IL-17A and IL-17F can induce the production of neutrophil, chemoattractants (CXCL1, CXCL2 and CXCL5) in epithelial cells^8,53^.

The relationship found in our study between the CMCs dis-functionality, with respect to the Th17-related cytokines, and effector functions exerted by these cytokines such as the induction of neutrophil recruiting chemokines, it is possible to have relevant consequences in the context of the local genital immunity. Thus, the lower levels of CXCL5 and CXCL1 found in HIV+ ectocervix samples could imply an impaired immunity against potential pathogens of the mucosal genital tract. In fact, relevance of IL-17 mediated immunity in the female genital tract has been analyzed in different studies against different pathogens. Accordingly, in one of them, a critical role for Th17 and IL-17 against *Neiserria gonorrheae* mice genital infection mediated by influx of neutrophils was demonstrated^54^, and another study showed that IL-17 contributes to the development of Th1 immunity and neutrophil recruitment during *Chlamidia muridarum* genital tract infection^55^. Even more, a recent study showed the importance of IL-17 in antiviral immunity in the female genital tract against HSV-2^56^.

We also detected higher levels of CCL17 (Treg chemoatractant^57,58^) and higher values in the ratio of CCL17/CCL20 (Th17 chemokine) in both HIV+ groups, which correlated directly with IL-10 levels and indirectly with IL-17A, respectively (Fig. 5III and IV). These data could suggest a trend to an immunoregulatory skew in the chemokine pattern in the genital samples of HIV+ women. Indeed, these results are in line with the altered genital mucosal Th17 functionality and minor alterations observed in Treg Cytokines (IL-10 and TFG-β) found in HIV+ ART naïve and treated individuals. Similar results were found in previous findings based on intestinal mucosal cells, where one of them showed a reduction in CCL20 production by intestinal cells from HIV+ treated individuals, with an increase in the frequency of gut FoxP3 Treg cells associated with a shift from IL-17 to IL-10 and TFG-β^59^. In another study, it was shown that the mucosal Th17 function in the intestine is altered during HIV infection, and the authors described an immunoregulatory skewing of Th17 function characterized by an increase in the IL-10/TNF-α ratio^28^.

It should be noted that despite the novelty of our findings, one of the constraints of the present study was that although Th17 and Treg-related cytokines secreted by CMCs were evaluated after PMA/Ionomycin stimulation (inducing principally T-cell activation) NK cells and other innate lymphoid cells can secrete IL-17A and IL-22, as well as macrophages can produce IL-10. On the other hand, another limitation is that although samples from patients with evidence of sexually transmitted infections (STIs) (associated with visible discharge, ulceration or macroscopic cervical changes) were excluded from the study, frequencies of actual lab-confirmed STIs or BV may have been underestimated, which may influence Th17 responses and cytokines.

CD161+ CD4+ T-cells, precursors of the Th17-cell population with gut-homing potential^46,47^, were found in significant minor proportions in PBMCs in both HIV+ groups compared to healthy donors, and to remark the levels of these cells in HIV+ ART+ did not result significantly higher than those observed in the group of HIV+ ART naïve individuals. Results from previous reports on CD161+ CD4+ T-cells in PBMCs of HIV+ subjects described a significant reduction in these cells of HIV-infected persons^16^, whereas another study did not find any differences in blood CD4+ CD161+ T-cells between HIV-negative and HIV-positive female sex-workers^52^. The positive correlations that we found between the proportions of CD4+ CD161+ T-cells in PBMCs and the levels of IL-17F and IL-21 production by CMCs from endocervical samples, may suggest an indication of CD4+ CD161+ T-cell homing to the mucosal genital tract, as it has recently been described for the first time that CD161+ CD4+ T cells are enriched in this mucosal site^52^. On the other hand, as this T-cell population has been identified with gut-homing characteristics, it may also suggest that a better homing of CD4+ CD161+ T-cells to the intestine also implies an improved restoration of these cells in the female genital tract (higher production of IL-21 and IL-17F cytokines from CMCs).

In brief, results from this study indicated that after ART, the altered Th17 and Th17/Treg proportions were normalized in peripheral blood. However, in FGT, abnormal patterns of secretion of Th17-related cytokines were observed in CMCs from HIV+ women even in those of the HIV+ ART+ group. This altered Th17-cytokines pattern, was associated with diminished levels of the CXCL5 and CXCL1 neutrophil chemokines and with an immunoregulatory skew in the ratio of CCL17/CCL20 in the ectocervix samples of these women. ART could not restore proportions of CD4+ CD161+ T-cells in PBMCs, and positive correlations between these cells and the levels of IL-17F and IL-21 production by CMCs may suggest that a better homing of CD4+ CD161+ T-cells to the intestine would also imply a better restoration of these cells in the female genital tract. The main findings of this study suggested that HIV infection has an impact on Th17-related functions at the female mucosal tract and ART initiated during chronic stage was not completely effective in restoring it.

## Materials and Methods

### Description of individuals included in the study

A total of 106 individuals participated in this study: 41 healthy HIV seronegative donors (HIV-negative) and 65 HIV infected patients of whom 33 were chronically infected patients *naïve* of antiretroviral therapy (HIV+ ART−) and 32 were chronically infected patients on antiretroviral therapy (HIV+ ART+). The main clinical characteristics of the subjects are described in Table 1. A portion (n = 20) of the total HIV-neg (n = 41) subjects included in this study were voluntary blood donors from *Sanatorio Dr*. *Julio Mendez* blood bank (Buenos Aires, Argentina; all of them were individuals older than 18 years of age that completed and passed a survey on blood donation and were screened for serological markers before being accepted as donors). The rest of them were recruited at the *Hospital General de Agudos* “*Dr*. *Juan A*. *Fernández*” [Buenos Aires, Argentina; all blood samples were confirmed to be negative for HIV-1 and HIV-2 by VIKIA HIV1/2 quick test (BioMérieux SA) followed by Genscreen ULTRA HIV Ag-Ab ELISA (BIO-RAD) according to the current diagnostic algorithm]. Chronic HIV+ ART− patients were defined as individuals with documented HIV-1 infection for more than 3 years or with CD4 counts less than 350, and with detectable plasma viral loads (VL; between 500 and 500.000 HIV-1 RNA copies/ml plasma), and ART *naïve* at the time of sample collection. Chronic HIV+ ART+ includes subjects with established HIV-1 infection for more than 3 years, with a median (IQR) of 14 (6.75–19) years from the first positive serology and were on ART. The main characteristics of the subjects of the different groups are described in Table 1. This study was reviewed and approved by two institutional review boards: *Comité de Ética en Investigación* Hospital Fernández (CEIHF) *Hospital General de Agudos Juan A*. *Fernández* (*Buenos Aires*, *Argentina; protocolo CODEI* 201115, 22/09/2014) and *Comité de Etica Fundación Huésped* (10/2013) (*Buenos Aires*, *Argentina*). All participants provided written informed consent and accepted to participate in this study. The methods applied were carried out in line with the approved guidelines.

### Collection and processing of blood samples

Blood samples were collected at enrollment on tubes with EDTA and centrifuged to separate plasma. Peripheral blood mononuclear cells (PBMCs) were isolated by Ficoll-Hypaque density gradient centrifugation (Amersham, Sweden) and cryopreserved by standard procedures. Plasma VL (branched-DNA, Versant HIV-1 RNA 3.0 assay; Siemens Healthcare, UK) and CD4/CD8 counts (flow cytometry double platform, BD FACSCanto; BD Biosciences) were routinely determined in samples from all HIV infected patients and in samples from the HIV-neg donors enrolled at the *Hospital General de Agudos* “*Dr*. *Juan A*. *Fernández*”. Subsequent functional assays were performed according to sample availability, using only thawed cells with >95% viability after overnight rest in complete RPMI medium [RPMI-1640 (Sigma-Aldrich, USA) supplemented with 10% fetal bovine serum (FBS; Gibco, USA), 2 mM L-glutamine (Sigma-Aldrich, USA), 100 U/ml penicillin (Sigma-Aldrich, USA), 100 mg/ml streptomycin (Sigma-Aldrich, USA), and 10 mM HEPES (Gibco, USA)].

### Collection and processing of female genital mucosal samples

Paired blood and genital mucosal samples were obtained from the women recruited in the study. Those female mucosal samples of women who were menstruating at sampling time, were peri or post-menopausal, or had undergone a hysterectomy were excluded from the study. Samples from patients with evidence of sexually transmitted infections (STIs) (associated with visible discharge, ulceration or macroscopic cervical changes) were also excluded. Considering the above mentioned variables to select the mucosal samples, the number of women that were screened to get the final number enrolled in each group were: 50 women for the HIV+ ART+ group (to get the 31 selected), approximately 27 women for the HIV− group (to get the 21 selected) and 19 women for the HIV+ ART− group (to get the 12 selected).

Female mucosal samples involved the collection of two types of samples in the following order: (i) under speculum examination, after cleaning the zone of excessive mucus, a cotton genital swab was introduced into the fornix, rotated 360° and immediately disposed in a 15 ml tube containing 3 ml of cold sterile PBS supplemented with 30 ul of a 100X cocktail of protease inhibitors (Cat Num: 78410 Thermo-Fischer) (ectocervical swab); (ii) Immediately afterwards, two cytobrushes (Digene Corp., USA) were sequentially introduced into the endocervical os, up to the transformation zone, rotated once 360° and immediately disposed in a single 15 ml tube containing 3 ml of cold RPMI medium [RPMI-1640 (Sigma-Aldrich, USA) supplemented with 2 mM L-glutamine (Sigma-Aldrich, USA), 100 U/ml penicillin (Sigma-Aldrich, USA), 100 mg/ml streptomycin (Sigma-Aldrich, USA), 10 mM HEPES (Gibco, USA) and 1% fungizone (Invitrogen)]. All samples were transported on ice and processed within 4 hours of collection. Samples with visible blood contamination and/or excessive mucus were discarded.

To obtain ectocervix soluble mediators, swab samples were processed as described previously^60^. The tube was vortexed for approximately 30 seconds and then the swab was compressed against the side of the tube to maximize elution of genital tract secretions. Afterwards, the swab was discarded and the tube was centrifuged for 10 minutes at 1500 rpm to remove cell debris. The supernatant was stored at −80 °C until use. Cervical mononuclear cells (CMCs) from the cytobrushes were obtained as previously described^21,61^, by manual shaking and extensive washing with a Pasteur pipette and filtered through a 100-mm filter (Becton Dickinson [BD]). After centrifugation for 10 min at 1500 rpm cell pellets were resuspended in complete RPMI medium supplemented with fungizone (1%). Viable CMCs were assessed by tripan blue dye and cells were immediately used. In preliminary experiments a phenotypical characterization of the CMCs obtained from the cytobrushes was performed, verifying that the proportions of CD3+, CD4+ and CD8-T cells and CD4:CD8 ratio found in CMCs samples obtained HIV-neg women were comparable to those values reported previously^62,63^(See Supplementary Table S5). Although few CMCs samples from HIV+ ART+ women could be characterized, proportions of CD3+, CD4+ and CD8-T cells were similar to those found in HIV-neg (Table S5).

### Determinations in PBMCs

#### Proportions of Th17 and Treg cells

Characterization of these T-cell subsets was performed by flow cytometry following methods described previously by our group^18^, using thawed and overnight rested PBMCs dispensed in U-bottom 96-well plates (between 5 × 10^5^ and 10^6^ cells/well were used, depending on sample availability). Th17 cells (defined as CD3+ CD4+ IL-17+) were identified by intracellular cytokine staining (ICS) after 6 hs of polyclonal stimulation at 37 °C with anti-CD3 and anti-CD28 Abs (10 ng/ml each) and monensin (0,7 μl/ml; GolgiStop, BD Biosciences). Cell viability and surface cell staining consisted of 30 min incubation at 4 °C with LIVE/DEAD Fixable NEAR-IR (Invitrogen) and anti-CD3-PECy7 plus anti-CD4-PerCP respectively. Then, ICS was performed following the instructions of the Cytofix/Cytoperm kit (BD Biosciences), incubated for 30 min at 4 °C with anti-IL-17A-PE. Unstimulated controls (medium only) were also included. Treg cells (defined as CD4+ CD25+ FoxP3+) were evaluated in unstimulated PBMCs, stained for 30 min at 4 °C with LIVE/DEAD Fixable NEAR-IR (Invitrogen), anti-CD3-PerCP, anti-CD4-FITC, anti-CD25-APC and anti-CD39-PECy7. For intranuclear staining, the Human FoxP3 Staining Kit (BD Biosciences) was used according to manufacturer’s instructions. Briefly, after the fixation and permeabilization steps, cells were incubated for 30 min at 4 °C with anti-FoxP3-PE. Isotype-matched APC- and PE-conjugated non-specific Abs were used in each sample to accurately set FoxP3 and CD25 negative populations.

#### T-cell activation proportions

Immune activation was defined as the percentage of CD38+ HLA-DR+ T-cells (CD4 or CD8) and analyzed by flow cytometry. For this, thawed and overnight rested PBMCs were surface stained for 30 min at 4 °C with LIVE/DEAD Fixable NEAR-IR (Invitrogen), anti-CD3-PeCy7, anti-CD4-PerCP, anti-CD8-PE, anti-CD38-APC and anti-HLA-DR-FITC. Isotype-matched FITC- and APC-conjugated non-specific Abs were used in each sample to set CD38 and HLA-DR negative populations.

#### Proportions of CD4+ CD161+ cells

Characterization of CD3+ CD4+ CD161+ cells was performed following the methodology described previously, briefly thawed and overnight rested PBMCs were surface stained during 30 min incubation at 4 °C with LIVE/DEAD Fixable NEAR-IR (Invitrogen), anti-CD3-PECy7, anti-CD4-PerCP and anti-CD161-APC.

All fluorochrome-conjugated, isotype and co-stimulatory antibodies (Abs) used in this study were obtained from BD Biosciences (USA).

### Gating Strategy

All samples were acquired in a 2-laser, 6-color BD FACSCanto flow cytometer and analyses were performed using the BD FACSDiva software. Instrument settings and fluorescence compensation were performed for each day of testing using unstained and single-stained samples. The same initial gating strategy was applied in all flow cytometry assays. The gating strategy employed is shown and described in Supplementary Fig. S6.

### Determinations in mucosal genital samples

#### Specific soluble mediators and total protein amounts in ectocervix samples

Simultaneous determination of the following 13 chemokines was performed in swab ectocervical supernatants using Cytometric Bead Assay (CBA Multi-Analyte Flow Assay Kit, Human Proinflammatory Chemokine Panel, BioLegend Inc., USA): IL-8, IP-10, Eotaxin, TARC, MCP-1, RANTES, MIP-1α, MIG, ENA-78, MIP-3α, GROα, I-TAC and MIP-1β. The limit of detection of this assay ranged between 1 and 2.7 pg/ml. Samples were acquired in a 2-laser, 6-color BD FACSCanto flow cytometer and analyses were performed using LEGEND Plex^TM^ Data Analysis Software v7.0 (VigeneTech, USA).

Determination of total protein amounts was performed in swab ectocervical supernatants using Micro BCA Protein Assay (Thermo Scientific) following the manufacturer´s instructions.

#### Stimulation of CMCs and cytokine quantification in culture supernatants

Viable CMCs obtained from the cytobrushes were resuspended in complete RPMI medium, dispensed in U-bottom 96-well plates (1–2 × 10^5^/200 μl) and stimulated with PMA/Ionomycin (Sigma-Aldrich; 10 ng/ml and 500 ng/ml, respectively) for 5 hs at 37 °C. After incubation, cells were centrifuged for 3 min at 4000 rpm and supernatants were stored at −80 °C until cytokine quantification. Simultaneous determination of IL-17A, IL-17F, IL-21 and IL-10 was performed in supernatants using CBA Mix and Match panel (BD Biosciences, USA), whereas IL-17A, IL-17F, IL-21, IL-22 and IL-10 was determined using the CBA Mix and Match panel (BioLegend, USA). Samples were acquired in a 2-laser, 6-color BD FACSCanto flow cytometer and analyses were performed using FCAP Array Software v3.0 (BD Biosciences, USA) or LEGEND Plex^TM^ Data Analysis Software v7.0 (VigeneTech, USA). Additionally, TGF-β1 was determined in CMCs supernatants using Free Active TGF-β1 ELISA Kit (BioLegend Inc., USA) following manufacturer’s instructions.

### Statistical analysis

The normality of all the variables was analyzed with the Shapiro-Wilk test. Mann-Whitney U two-sided test was used for nonparametric comparisons. Pearson or Spearman´s rank tests were used for correlations with normal or non-normal distribution respectively. Comparisons between proportions were done with Chi-Square test. For correlations involving cytokine levels, *p‐*values were adjusted for multiple comparisons using a false discovery rate (FDR) procedure, according to the original FDR method of Benjamini and Hochberg.

All statistical analyses were performed using GraphPad Prism 7.00 (GraphPad Software, USA). Two sample t-tests between percentages were calculated using Statstics Calculator (StatPac Inc., USA). All tests were considered significant if the p value obtained was less than 0.05.

## Supplementary information

Supplementary Information

## Data Availability

All data generated or analyzed during this study are included in this published article (and its Supplementary Information Files).

## References

[1] Valverde-Villegas JM, Matte MC, de Medeiros RM, Chies JA (2015). New Insights about Treg and Th17 Cells in HIV Infection and Disease Progression. J Immunol Res.

[2] Pandiyan P (2016). Mucosal Regulatory T Cells and T Helper 17 Cells in HIV-Associated Immune Activation. Frontiers in immunology.

[3] Ouyang W, Kolls JK, Zheng Y (2008). The biological functions of T helper 17 cell effector cytokines in inflammation. Immunity.

[4] Spolski R, Leonard WJ (2009). Cytokine mediators of Th17 function. European journal of immunology.

[5] Blaschitz C, Raffatellu M (2010). Th17 cytokines and the gut mucosal barrier. Journal of clinical immunology.

[6] Cook DN (2000). CCR6 mediates dendritic cell localization, lymphocyte homeostasis, and immune responses in mucosal tissue. Immunity.

[7] Kolls JK, Linden A (2004). Interleukin-17 family members and inflammation. Immunity.

[8] Onishi RM, Gaffen SL (2010). Interleukin-17 and its target genes: mechanisms of interleukin-17 function in disease. Immunology.

[9] Wu W, Chen F, Liu Z, Cong Y (2016). Microbiota-specific Th17 Cells: Yin and Yang in Regulation of Inflammatory Bowel Disease. Inflamm Bowel Dis.

[10] Chen Y (2003). Stimulation of airway mucin gene expression by interleukin (IL)-17 through IL-6 paracrine/autocrine loop. J Biol Chem.

[11] Pandiyan P, Zheng L, Lenardo MJ (2011). The molecular mechanisms of regulatory T cell immunosuppression. Frontiers in immunology.

[12] Pandiyan P, Zheng L, Ishihara S, Reed J, Lenardo MJ (2007). CD4+ CD25+ Foxp3+ regulatory T cells induce cytokine deprivation-mediated apoptosis of effector CD4+ T cells. Nat Immunol.

[13] Fazekas de St Groth B, Landay AL (2008). Regulatory T cells in HIV infection: pathogenic or protective participants in the immune response?. Aids.

[14] Chevalier MF, Weiss L (2013). The split personality of regulatory T cells in HIV infection. Blood.

[15] Brenchley JM (2008). Differential Th17 CD4 T-cell depletion in pathogenic and nonpathogenic lentiviral infections. Blood.

[16] Prendergast A (2010). HIV-1 infection is characterized by profound depletion of CD161+ Th17 cells and gradual decline in regulatory T cells. Aids.

[17] Kanwar B, Favre D, McCune JM (2010). Th17 and regulatory T cells: implications for AIDS pathogenesis. Current opinion in HIV and AIDS.

[18] Falivene J (2015). Th17 and Th17/Treg ratio at early HIV infection associate with protective HIV-specific CD8(+) T-cell responses and disease progression. Scientific reports.

[19] Abel K, Rocke DM, Chohan B, Fritts L, Miller CJ (2005). Temporal and anatomic relationship between virus replication and cytokine gene expression after vaginal simian immunodeficiency virus infection. Journal of virology.

[20] Haase AT (2011). Early events in sexual transmission of HIV and SIV and opportunities for interventions. Annual review of medicine.

[21] McKinnon LR (2011). Characterization of a human cervical CD4+ T cell subset coexpressing multiple markers of HIV susceptibility. Journal of immunology.

[22] McKinnon LR (2015). Early HIV-1 infection is associated with reduced frequencies of cervical Th17 cells. Journal of acquired immune deficiency syndromes.

[23] Stieh DJ (2016). Th17 Cells Are Preferentially Infected Very Early after Vaginal Transmission of SIV in Macaques. Cell host & microbe.

[24] Chege D (2012). Blunted IL17/IL22 and pro-inflammatory cytokine responses in the genital tract and blood of HIV-exposed, seronegative female sex workers in Kenya. Plos One.

[25] Gosselin A (2010). Peripheral blood CCR4+ CCR6+ and CXCR3+ CCR6+ CD4+ T cells are highly permissive to HIV-1 infection. Journal of immunology.

[26] Macal M (2008). Effective CD4+ T-cell restoration in gut-associated lymphoid tissue of HIV-infected patients is associated with enhanced Th17 cells and polyfunctional HIV-specific T-cell responses. Mucosal immunology.

[27] Mehandru S (2006). Lack of mucosal immune reconstitution during prolonged treatment of acute and early HIV-1 infection. Plos medicine.

[28] Kim CJ (2013). Mucosal Th17 cell function is altered during HIV infection and is an independent predictor of systemic immune activation. Journal of immunology.

[29] Schuetz A (2014). Initiation of ART during early acute HIV infection preserves mucosal Th17 function and reverses HIV-related immune activation. Plos pathogens.

[30] Zeitz M, Ullrich R, Schneider T, Schieferdecker HL, Riecken EO (1994). Cell differentiation and proliferation in the gastrointestinal tract with respect to the local immune system. Ann N Y Acad Sci.

[31] Nilsson J (2006). HIV-1-driven regulatory T-cell accumulation in lymphoid tissues is associated with disease progression in HIV/AIDS. Blood.

[32] Shaw JM (2011). Increased frequency of regulatory T cells accompanies increased immune activation in rectal mucosae of HIV-positive noncontrollers. Journal of virology.

[33] Saito S (2007). Regulatory T cells and regulatory natural killer (NK) cells play important roles in feto-maternal tolerance. Semin Immunopathol.

[34] Shacklett BL (2009). Cell-mediated immunity to HIV in the female reproductive tract. J Reprod Immunol.

[35] Thibodeau V (2017). Highly-Exposed HIV-1 seronegative Female Commercial Sex Workers sustain in their genital mucosa increased frequencies of tolerogenic myeloid and regulatory T-cells. Scientific reports.

[36] Rodriguez-Garcia M, Barr FD, Crist SG, Fahey JV, Wira CR (2014). Phenotype and susceptibility to HIV infection of CD4+ Th17 cells in the human female reproductive tract. Mucosal immunology.

[37] Masson L (2015). Relationship between female genital tract infections, mucosal interleukin-17 production and local T helper type 17 cells. Immunology.

[38] Chevalier MF (2013). The Th17/Treg ratio, IL-1RA and sCD14 levels in primary HIV infection predict the T-cell activation set point in the absence of systemic microbial translocation. Plos pathogens.

[39] Favre D (2010). Tryptophan catabolism by indoleamine 2,3-dioxygenase 1 alters the balance of TH17 to regulatory T cells in HIV disease. Science translational medicine.

[40] Zheng L (2014). Factors associated with CD8+ T-cell activation in HIV-1-infected patients on long-term antiretroviral therapy. Journal of acquired immune deficiency syndromes.

[41] Gandhi RT (2006). Effect of baseline- and treatment-related factors on immunologic recovery after initiation of antiretroviral therapy in HIV-1-positive subjects: results from ACTG 384. Journal of acquired immune deficiency syndromes.

[42] Zhao H, Bo C, Kang Y, Li H (2017). What Else Can CD39 Tell Us?. Frontiers in immunology.

[43] Bebell LM (2008). Relationship between levels of inflammatory cytokines in the genital tract and CD4+ cell counts in women with acute HIV-1 infection. The Journal of infectious diseases.

[44] Masson L (2015). Genital inflammation and the risk of HIV acquisition in women. Clinical infectious diseases: an official publication of the Infectious Diseases Society of America.

[45] Griffith JW, Sokol CL, Luster AD (2014). Chemokines and chemokine receptors: positioning cells for host defense and immunity. Annu Rev Immunol.

[46] Cosmi L (2008). Human interleukin 17-producing cells originate from a CD161+ CD4+ T cell precursor. The Journal of experimental medicine.

[47] Kleinschek MA (2009). Circulating and gut-resident human Th17 cells express CD161 and promote intestinal inflammation. The Journal of experimental medicine.

[48] Peng Q (2013). Imbalances of gut-homing CD4+ T-cell subsets in HIV-1-infected Chinese patients. Journal of acquired immune deficiency syndromes.

[49] He Y (2012). A randomized case-control study of dynamic changes in peripheral blood Th17/Treg cell balance and interleukin-17 levels in highly active antiretroviral-treated HIV type 1/AIDS patients. AIDS research and human retroviruses.

[50] Jenabian MA, Ancuta P, Gilmore N, Routy JP (2012). Regulatory T cells in HIV infection: can immunotherapy regulate the regulator?. Clinical & developmental immunology.

[51] Seddiki N (2014). Human antigen-specific CD4(+) CD25(+) CD134(+) CD39(+) T cells are enriched for regulatory T cells and comprise a substantial proportion of recall responses. European journal of immunology.

[52] Boily-Larouche G (2017). CD161 identifies polyfunctional Th1/Th17 cells in the genital mucosa that are depleted in HIV-infected female sex workers from Nairobi, Kenya. Scientific reports.

[53] Zhang M, Wang G, Tao Y, Zhang H (2015). The proinflammatory effect and molecular mechanism of IL- 17 in the intestinal epithelial cell line HT-29. Journal of B.U.ON.: official journal of the Balkan Union of Oncology.

[54] Feinen B, Jerse AE, Gaffen SL, Russell MW (2010). Critical role of Th17 responses in a murine model of Neisseria gonorrhoeae genital infection. Mucosal immunology.

[55] Scurlock AM (2011). Interleukin-17 contributes to generation of Th1 immunity and neutrophil recruitment during Chlamydia muridarum genital tract infection but is not required for macrophage influx or normal resolution of infection. Infection and immunity.

[56] Bagri, P. *et al*. Novel Role for Interleukin-17 in Enhancing Type 1 Helper T Cell Immunity in the Female Genital Tract following Mucosal Herpes Simplex Virus 2 Vaccination. *Journal of virology* **91**, 10.1128/JVI.01234-17 (2017). 10.1128/JVI.01234-17 PMC5686749 28956763

[57] Osabe M, Tajika T, Tohkin M (2018). Allopurinol suppresses expression of the regulatory T-cell migration factors TARC/CCL17 and MDC/CCL22 in HaCaT keratinocytes via restriction of nuclear factor-kappaB activation. J Appl Toxicol.

[58] Ishida T, Ueda R (2006). CCR4 as a novel molecular target for immunotherapy of cancer. Cancer Sci.

[59] Loiseau C (2016). CCR6(−) regulatory T cells blunt the restoration of gut Th17 cells along the CCR6-CCL20 axis in treated HIV-1-infected individuals. Mucosal immunology.

[60] Dezzutti CS (2011). Performance of swabs, lavage, and diluents to quantify biomarkers of female genital tract soluble mucosal mediators. Plos One.

[61] Bere A, Denny L, Hanekom W, Burgers WA, Passmore JA (2010). Comparison of polyclonal expansion methods to improve the recovery of cervical cytobrush-derived T cells from the female genital tract of HIV-infected women. Journal of immunological methods.

[62] Nkwanyana NN (2009). Impact of human immunodeficiency virus 1 infection and inflammation on the composition and yield of cervical mononuclear cells in the female genital tract. Immunology.

[63] Prakash M, Patterson S, Kapembwa MS (2001). Macrophages are increased in cervical epithelium of women with cervicitis. Sexually transmitted infections.

