# Supplementary material for: Impact of HIV-ART on the restoration of Th17 and Treg cells in blood and female genital mucosa
Source: Sci Rep. 2019 Feb 13;9:1978. doi: 10.1038/s41598-019-38547-1 (PMC6374372; doi:10.1038/s41598-019-38547-1)

SUPPLEMENTARY INFO:

Impact of HIV-ART on the restoration of Th17 and Treg cells in blood and female genital mucosa

María Paula Caruso[1#], Juliana Falivene[1#], María Pía Holgado[1], Diego Hernán Zurita[2], Natalia Laufer[1,2], Carina Castro[3], Ángeles Nico[2], Cynthia Maeto[1],Jimena Salido[1], Héctor Pérez[2], Horacio Salomón[1], Pedro Cahn[2,3] , Omar Sued[3], Valeria Fink[2,3], Gabriela Turk[1], María Magdalena Gherardi[1]*.

[1]Instituto de Investigaciones Biomédicas en Retrovirus y SIDA (INBIRS), Universidad de Buenos Aires-CONICET, Buenos Aires, Argentina. [2]Hospital J.A. Fernández, Buenos Aires, Argentina. [3]Fundación Huésped, Buenos Aires, Argentina. [3]Centro Medico Accord, Buenos Aires, Argentina.

RUNNING TITLE: HIV-ART effects on Th17 and Treg cells in the female genital tract

[#]Both authors contributed equally to the realization of this work

[*]**CORRESPONDING AUTHOR**

Dr. M. Magdalena Gherardi
Instituto de Investigaciones Biomédicas en Retrovirus y SIDA INBIRS (ex Centro Nacional de Referencia para el SIDA), Universidad de Buenos Aires- CONICET
Paraguay 2155 Piso 11
C1121ABG - Buenos Aires, Argentina
TE +54 11 4508 3689 ext 129
FAX +54 11 4508 3705
e-mail: mgherardi@fmed.uba.ar; mmgherardi@gmail.com

<u>Supplementary TABLE S1</u>: Details of the antiretroviral treatment of each of the individuals of the HIV+ART+ group.

Individual	Antiretroviral Regime
HIV+ ART+ 1	ABC 3TC NVP
HIV+ ART+ 2	ATV/r 3TC AZT
HIV+ ART+ 3	ATV/r 3TC AZT
HIV+ ART+ 4	LPV/r FTC TDF
HIV+ ART+ 5	3TC LPV/r ABC
HIV+ ART+ 6	EFV 3TC
HIV+ ART+ 7	3TC TDF EFV
HIV+ ART+ 8	ABC 3TC NVP
HIV+ ART+ 9	ABC 3TC ATV RTV
HIV+ ART+ 10	TDF 3TC ATV RTV
HIV+ ART+ 11	no data
HIV+ ART+ 12	DRV RTV RAL
HIV+ ART+ 13	TDF 3TC LPV/r
HIV+ ART+ 14	FTC TDF ATV RTV
HIV+ ART+ 15	TDF 3TC EFV
HIV+ ART+ 16	FTC TDF NVP
HIV+ ART+ 17	ABC 3TC EFV
HIV+ ART+ 18	AZT 3TC LPV/r
HIV+ ART+ 19	ABC 3TC NVP
HIV+ ART+ 20	FTC TDF RTV ATV
HIV+ ART+ 21	FTC TDF EFV
HIV+ ART+ 22	no data
HIV+ ART+ 23	no data
HIV+ ART+ 24	no data
HIV+ ART+ 25	no data
HIV+ ART+ 26	no data
HIV+ ART+ 27	no data
HIV+ ART+ 28	DRV/r 3TC AZT TDF
HIV+ ART+ 29	RAL MRV TDF
HIV+ ART+ 30	DRV/r RAL MRV
HIV+ ART+ 31	ATV 3TC AZT
HIV+ ART+ 32	EFV 3TC AZT

ART: antiretroviral treatment. ABC: abacavir. 3TC: lamivudine. NVP: nevirapine. ATV/r: atazanavir/ritonavir. AZT: zidovudine. LPV/r: lopinavir/ritonavir. FTC: emtricitabine. TDF: tenofovir disoproxil fumarate. EFV: efavirenz. ATV: atazanavir. RTV: ritonavir. DRV: darunavir. DRV/r: darunavir/ritonavir. RAL: raltegravir. MRV: maraviroc.

<u>Supplementary FIGURE S2</u>: Qualitative analysis of the Th17 and Treg-related cytokine responses in supernatants from stimulated Cervix Mononuclear Cells (CMCs). (A) Bars represent percentages of patients with positive (above limit of detection) cytokine responses, of Th17-related cks (left panel) and Treg related cks (right panel). (B) Bars represent the median number of the indicated cytokines secreted plus IQR of Th17-related cks (left panel) or Treg related cks (right panel). Significant differences between groups are depicted as *p<0.05, **p<0.01.

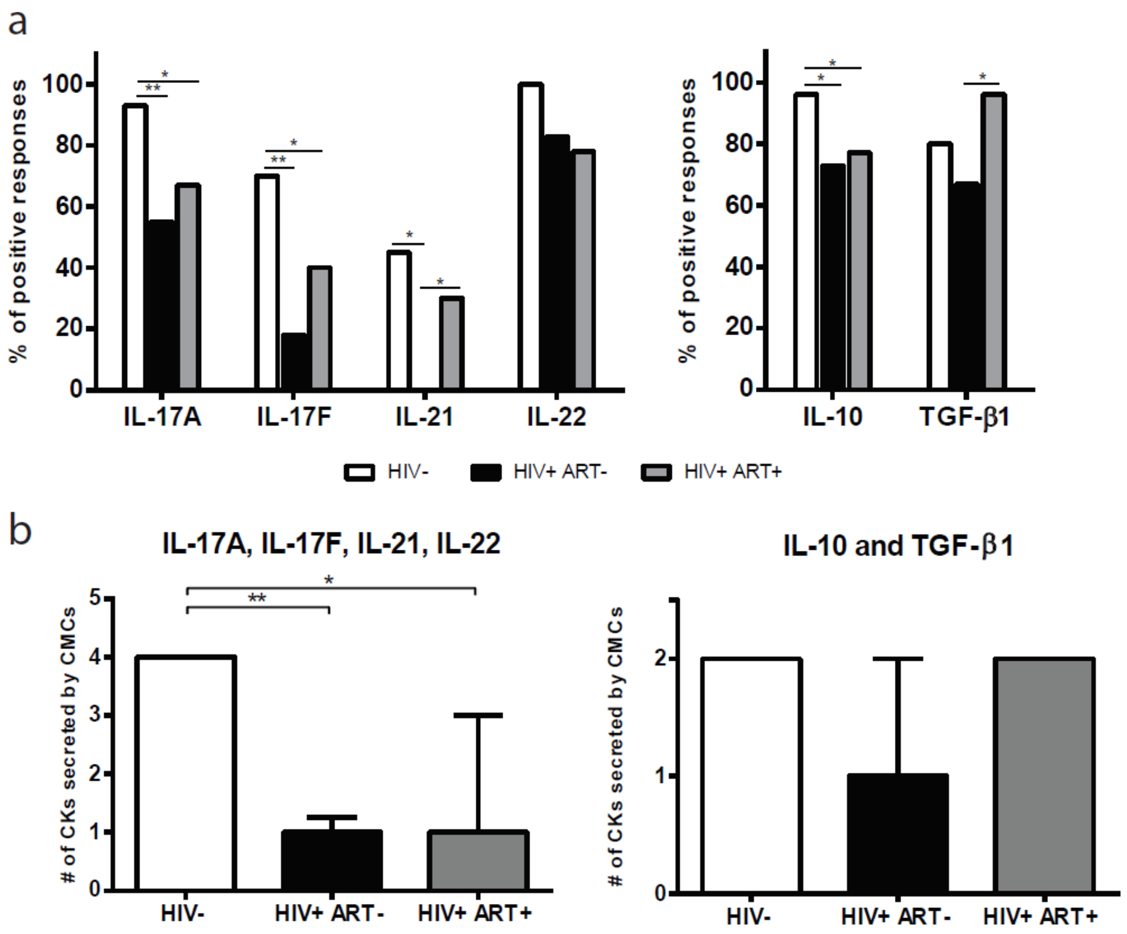

<u>Supplementary TABLE S3</u>: Analysis of chemokine quantities in the supernatants of ectocervix samples of the study groups. A total of thirteen chemokines were determined by the CBA method. ART: antiretroviral treatment. Data is shown as median (IQR) (IQR: inter quartile range). Significant differences in comparison to the HIV-neg group: *p<0.05, **p<0.01. No differences were found between HIV+ART- and HIV+ART+ groups.

CHEMOKINE (pg/ml)	HIV- n = 21	HIV+ ART- n = 12	HIV+ ART+ n = 31
IL-8	477.40 (147.10 - 830.80)	156.60 (22.35 - 1368.00)	200.70 (91.65 - 503.70)
IP-10	9.31 (4.60 - 21.27)	21.58 (6.39 - 76.55)	12.10 (3.70 - 39.33)
Eotaxin	3.78 (3.47 - 4.69)	4.69 (3.01 - 6.33)	4.18 (3.78 - 5.00)
TARC / CCL17	3.35 (2.49 - 4.06)	4.78 (3.00 - 6.43)*	4.60 (3.35 - 5.33)**
MCP-1	7.83 (3.73 - 19.49)	7.56 (3.58 - 51.78)	16.78 (5.56 - 38.84)
RANTES	6.23 (4.81 - 19.04)	11.39 (6.44 - 24.39)	6.64 (5.01 - 14.87)
MIP-1α	4.98 (4.44 - 9.89)	8.47 (4.98 - 25.11)	6.07 (4.71 - 7.95)
MIG	4.76 (2.60 - 16.59)	12.23 (4.88 - 59.17)	7.72 (3.28 - 17.84)
ENA-78 / CXCL5	51.34 (18.76 - 100.10)	12.89 (5.98 - 47.96)*	9.60 (4.96 - 28.31)**
MIP-3α / CCL20	4.98 (2.69 - 9.19)	3.69 (2.33 - 6.08)	3.17 (2.69 - 5.99)
GRO-α / CXCL1	149.00 (98.87 - 226.90)	60.28 (24.52 - 501.20)	69.87 (34.66 - 127.10)**
I-TAC	2.72 (2.07 - 3.93)	3.70 (2.09 - 6.77)	2.98 (2.19 - 4.27)
MIP-1ß	3.75 (3.06 - 7.07)	3.97 (2.85 - 9.23)	3.53 (2.69 - 4.61)

<u>Supplementary FIGURE S4</u>: Correlation of the CD4+ CD161+ T-cells in PBMCs with IL-21 production by genital CMCs. Correlations found in (a) HIV+ART+ and (b) HIV-neg patients: log %CD4+ CD161+ T-cells vs log IL-21. *r* and *p* values correspond to Spearman's correlations. Symbols represent individual patients: HIV-neg (○) and HIV+ ART+ (⊙).

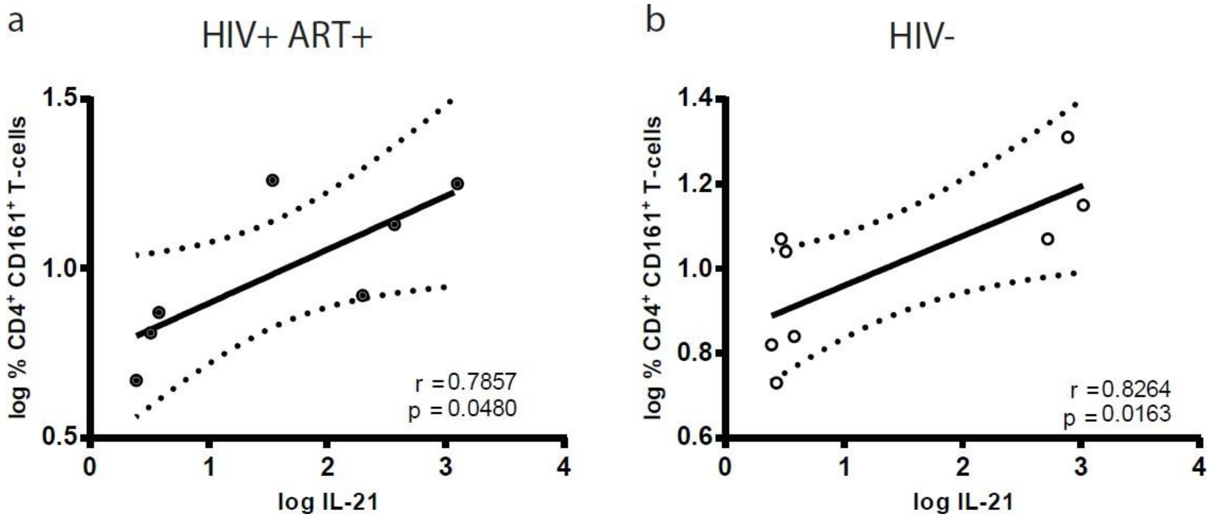

Supplementary TABLE S5: Phenotypical characteristics of CMCs obtained from cytobrushes of HIV-neg and HIV+ART´+ women.

IQR: inter quartile range.

GROUP	HIV-<br>(n = 16)	HIV+ ART+<br>(n = 3)
%CD3<br>median (IQR)	18.30 (1.69 - 28.74)	21.19 (1.2 - 41.18)
%CD4<br>median (IQR)	56.17 (34.90 - 71.93)	50.57 (1.7 - 96.29)
%CD8<br>median (IQR)	26.92 (15.24 - 36.77)	33.97 (0.34 - 51.18)
CD4/CD8 ratio<br>median (IQR)	1.76 (0.88 - 4.13)	1.49 (0.03 - 1.49)

<u>Supplementary FIGURE S6</u>: Gating Strategy. The same initial gating strategy was applied in all flow cytometry assays. First, a) FCS forward scatter area (FSC-A) vs. height (FSC-H) was constructed to remove doublets. Then, b) small lymphocytes were selected in a plot of FSC vs. side scatter (SSC). c) Dead cells were then excluded by the LIVE/DEAD fluorescence. Subsequently, d) CD3+ CD4+ and e) CD3+ CD8+ events were gated in a CD3 vs. CD4 or CD8 dot plot. f) For selection of Th17 cells and g) CD161+ cells, the corresponding CD4 vs. IL-17 and CD4 vs. CD161 dot plots were constructed. h) For Treg evaluation, CD4 vs. CD25 and CD4 vs. FoxP3 dot plots were constructed and the “derived gate tool” was used to accurately and automatically determine the double positive CD25+ FoxP3+ population. Also, CD4 vs. CD39 was constructed. i) For evaluation of T-cell activation, CD4 (or CD8) vs. CD38 and CD4 (or CD8) vs. HLA-DR dot plots were constructed and the “derived gate tool” was used to accurately and automatically determine the double positive CD38+ HLA-DR+ populations. (Between 500.000 and 100.000 events were acquired).

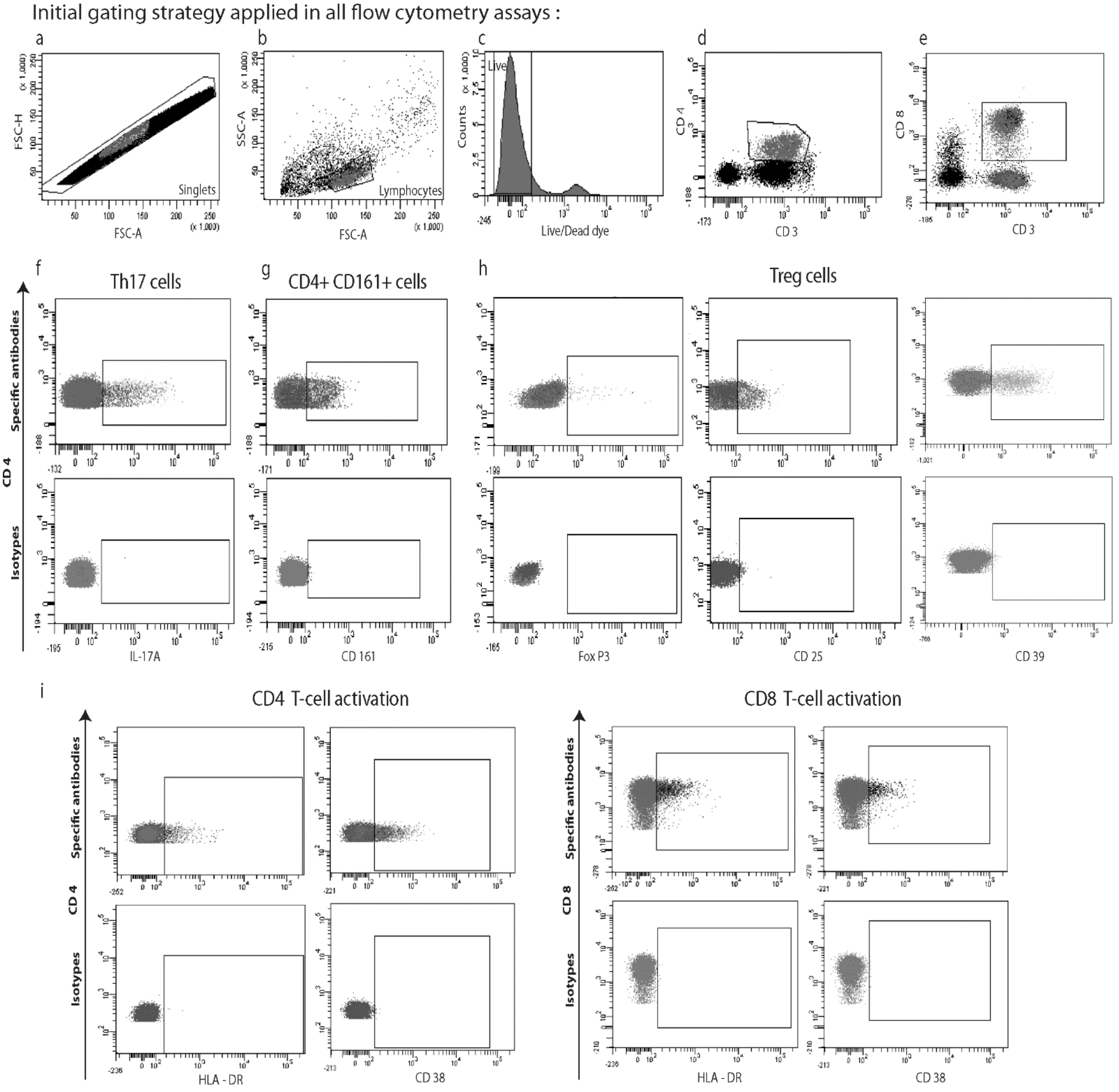

Supplement: Supplementary file 1 — Supplementary Information [file 41598_2019_38547_MOESM1_ESM.pdf]
